# Supplementary material for: Using social networks to scale up and sustain community-based programmes to improve physical activity and diet in low-income and middle-income countries: a scoping review protocol
Source: BMJ Open. 2021 Sep 14;11(9):e053586. doi: 10.1136/bmjopen-2021-053586 (PMC8442048; doi:10.1136/bmjopen-2021-053586)
Supplement: Supplementary data [file bmjopen-2021-053586supp001.pdf]

Supplementary additional file 1: Search strategy

PubMed (2021-05-10)

Concepts	Search strategy keywords	Search	# Results
Community-based programmes	(("community-based" OR "community based" OR community OR complex OR collective) AND (intervention OR program* OR trial OR evaluation OR action OR research)) OR ("Community-Based Participatory Research"[Mesh]) OR "Community Participation"[Mesh]	#1	2,174,786
Social networks	("social network*" OR "social network analysis" OR "social network intervention*" OR "network analysis" OR "community networks" OR “social relationships” OR “social capital” OR “whole networks”) OR (("Social Network Analysis"[Mesh] OR "Social Networking"[Mesh]) OR ("Community Networks"[Mesh]))	#2	56,178
Scale-up and sustainability	(("scale-up" OR "scale up" OR sustain* OR implement* OR disseminat*) OR (("Diffusion of Innovation"[Mesh]) OR "Program Evaluation"[Mesh])) OR ("diffusion of innovation" OR "program evaluation")	#3	1,194,227
Non-communicable disease prevention (physical activity and diet)	(Physical activity OR exercise OR diet OR "non communicable" OR chronic OR health promotion OR weight) OR ("Exercise"[Mesh] OR "Diet"[Mesh] OR "Noncommunicable Diseases"[Mesh] OR "Chronic Disease"[Mesh] OR "Health Promotion"[Mesh] OR "Obesity"[Mesh])	#4	4,006,384
Low- and middle-income country setting	((Afghanistan OR Albania OR Algeria OR American Samoa OR Angola OR Armenia OR Azerbaijan OR Bangladesh OR Belarus OR Byelarus OR Belorussia OR Belize OR Benin OR Bhutan OR Bolivia OR Bosnia OR Botswana OR Brazil OR Bulgaria OR Burma OR Burkina Faso OR Burundi OR Cabo Verde OR Cape Verde OR Cambodia OR Cameroon OR Central African Republic OR Chad OR China OR Colombia OR Comoros OR Comores OR Comoro OR Congo OR Costa Rica OR Côte d'Ivoire OR Cuba OR Djibouti OR Dominica OR Dominican Republic OR Ecuador OR Egypt OR El Salvador OR Equatorial Guinea OR Eritrea OR Ethiopia OR Fiji OR Gabon OR Gambia OR Gaza OR Georgia OR Georgia Republic OR Ghana OR Grenada OR Grenadines OR Guatemala OR Guinea OR Guinea-Bissau OR Guyana OR Haiti OR Herzegovina OR Hercegovina OR Honduras OR India OR Indonesia OR Iran OR Iraq OR Ivory Coast OR Jamaica OR Jordan OR Kazakhstan OR Kenya OR Kiribati OR Democratic People’s Republic of Korea OR Kosovo OR Kyrgyz OR	#5	6,170,610

	Kirghizia OR Kirghiz OR Kyrgyzstan OR Lao PDR OR Laos OR Lebanon OR Lesotho OR Liberia OR Libya OR Macedonia OR Madagascar OR Malawi OR Malay OR Malaya OR Malaysia OR Maldives OR Mali OR Marshall Islands OR Mauritania OR Mauritius OR Mexico OR Micronesia OR Moldova OR Mongolia OR Montenegro OR Morocco OR Mozambique OR Myanmar OR Namibia OR Nepal OR Nicaragua OR Niger OR Nigeria OR Pakistan OR Palau OR Papua New Guinea OR Paraguay OR Peru OR Philippines OR Principe OR Romania OR Ruanda OR Rwanda OR Samoa OR Sao Tome OR Senegal OR Serbia OR Sierra Leone OR Solomon Islands OR Somalia OR South Africa OR South Sudan OR Sri Lanka OR St Lucia OR St Vincent OR Sudan OR Surinam OR Suriname OR Swaziland OR Syria OR Syrian Arab Republic OR Tajikistan OR Tadzhikistan OR Tajikistan OR Tadzhik OR Tanzania OR Thailand OR Timor OR Togo OR Tonga OR Tunisia OR Turkey OR Turkmen OR Turkmenistan OR Tuvalu OR Uganda OR Ukraine OR Uzbek OR Uzbekistan OR Vanuatu OR Venezuela OR Vietnam OR West Bank OR Yemen OR Zambia OR Zimbabwe) OR (Deprived Countries OR Developing Countries OR Developing Country OR Developing Economies OR Developing Economy OR Developing Nation OR Developing Nations OR Developing World OR LAMI Countries OR LAMI Country OR Less Developed Countries OR Less Developed Country OR Less Developed Economies OR Less Developed Nation OR Less Developed Nations OR Less Developed World OR Lesser Developed Countries OR Lesser Developed Nations OR LMIC OR LMICS OR Low GDP OR Low GNP OR Low Gross Domestic OR Low Gross National OR Low Income OR Lower GDP OR lower gross domestic OR Lower Income OR Middle Income OR Poor Countries OR Poor Country OR Poor Economies OR Poor Economy OR Poor Nation OR Poor Nations OR Poor Population OR Poor Populations OR poor world OR Poorer Countries OR Poorer Economies OR Poorer Economy OR Poorer Nations OR Poorer Population OR Poorer Populations OR Third World OR Transitional Countries OR Transitional Country OR Transitional Economies OR Transitional Economy OR Under Developed Countries OR Under Developed Country OR under developed nations OR Under Developed World OR Under Served Population OR Under Served Populations OR Underdeveloped Countries OR Underdeveloped Country OR underdeveloped economies OR underdeveloped nations OR underdeveloped population OR Underdeveloped World OR Underserved Countries OR Underserved Nations OR Underserved Population OR Underserved Populations)) OR ("Developing Countries"[Mesh]) OR Global South OR Africa OR Latin America OR South America OR Sub-Saharan Africa OR Asia		

Search string	#1 AND #2 AND #3 AND #4 AND #5	#6	584
Limits	Limit to English language; Limit to since year 2000		
Total Result	#1 AND #2 AND #3 AND #4 AND #5 AND limits	#7	556

Scopus (2021-05-10)

Concepts	Search strategy keywords	Search	# Results
Community-based programmes	ALL (("community-based" OR "community based" OR community OR complex OR collective) AND (intervention OR program* OR trial OR evaluation OR action OR research))	#1	12,372,281
Social networks	ALL ("social network*" OR "social network analysis" OR "social network intervention*" OR "network analysis" OR "community networks" OR “social relationships” OR “social capital” OR “whole networks”)	#2	979,911
Scale-up and sustainability	ALL ("scale-up" OR "scale up" OR sustain* OR implement* OR disseminat* OR "diffusion of innovation" OR "program evaluation")	#3	9,528,545
Non-communicable disease prevention (physical activity and diet)	ALL (Physical activity OR exercise OR diet OR "non communicable" OR chronic OR health promotion OR weight)	#4	849,134
Low- and middle-income country setting	ALL ((Afghanistan OR Albania OR Algeria OR “American Samoa” OR Angola OR Armenia OR Azerbaijan OR Bangladesh OR Belarus OR Byelarus OR Belorussia OR Belize OR Benin OR Bhutan OR Bolivia OR Bosnia OR Botswana OR Brazil OR Bulgaria OR Burma OR “Burkina Faso” OR Burundi OR “Cabo Verde” OR “Cape Verde” OR Cambodia OR Cameroon OR “Central African Republic” OR Chad OR China OR Colombia OR Comoros OR Comores OR Comoro OR Congo OR “Costa Rica” OR “Côte d'Ivoire” OR Cuba OR Djibouti OR Dominica OR “Dominican Republic” OR Ecuador OR Egypt OR “El Salvador” OR “Equatorial Guinea” OR Eritrea OR Ethiopia OR Fiji OR Gabon OR Gambia OR Gaza OR Georgia OR “Georgia Republic” OR Ghana OR Grenada OR Grenadines OR Guatemala OR Guinea OR Guinea- Bissau OR Guyana OR Haiti OR Herzegovina OR Hercegovina OR Honduras OR India OR Indonesia OR Iran OR Iraq OR “Ivory Coast” OR Jamaica OR Jordan OR Kazakhstan OR Kenya OR Kiribati OR “Democratic People’s Republic of Korea” OR Kosovo OR Kyrgyz OR Kirghizia OR Kirghiz OR Kyrgyzstan OR Lao PDR OR Laos OR Lebanon OR Lesotho OR Liberia OR Libya OR Macedonia OR Madagascar OR Malawi OR	#5	5,148,871

	Malay OR Malaya OR Malaysia OR Maldives OR Mali OR “Marshall Islands” OR Mauritania OR Mauritius OR Mexico OR Micronesia OR Moldova OR Mongolia OR Montenegro OR Morocco OR Mozambique OR Myanmar OR Namibia OR Nepal OR Nicaragua OR Niger OR Nigeria OR Pakistan OR Palau OR “Papua New Guinea” OR Paraguay OR Peru OR Philippines OR Principe OR Romania OR Ruanda OR Rwanda OR Samoa OR “Sao Tome” OR Senegal OR Serbia OR “Sierra Leone” OR “Solomon Islands” OR Somalia OR “South Africa” OR “South Sudan” OR “Sri Lanka” OR “St Lucia” OR “St Vincent” OR Sudan OR Surinam OR Suriname OR Swaziland OR Syria OR “Syrian Arab Republic” OR Tajikistan OR Tadzhikistan OR Tajikistan OR Tadzhik OR Tanzania OR Thailand OR Timor OR Togo OR Tonga OR Tunisia OR Turkey OR Turkmen OR Turkmenistan OR Tuvalu OR Uganda OR Ukraine OR Uzbek OR Uzbekistan OR Vanuatu OR Venezuela OR Vietnam OR “West Bank” OR Yemen OR Zambia OR Zimbabwe) OR (“Deprived Countries” OR “Developing Countries” OR “Developing Country” OR “Developing Economies” OR “Developing Economy” OR “Developing Nation” OR “Developing Nations” OR “Developing World” OR “LAMI Countries” OR “LAMI Country” OR “Less Developed Countries” OR “Less Developed Country” OR “Less Developed Economies” OR “Less Developed Nation” OR “Less Developed Nations” OR “Less Developed World” OR “Lesser Developed Countries” OR “Lesser Developed Nations” OR LMIC OR LMICS OR “Low GDP” OR “Low GNP” OR “Low Gross Domestic” OR “Low Gross National” OR “Low Income” OR “Lower GDP” OR “lower gross domestic” OR “Lower Income” OR “Middle Income” OR “Poor Countries” OR “Poor Country” OR “Poor Economies” OR “Poor Economy” OR “Poor Nation” OR “Poor Nations” OR “Poor Population” OR “Poor Populations” OR “poor world” OR “Poorer Countries” OR “Poorer Economies” OR “Poorer Economy” OR “Poorer Nations” OR “Poorer Population” OR “Poorer Populations” OR “Third World” OR “Transitional Countries” OR “Transitional Country” OR “Transitional Economies” OR “Transitional Economy” OR “Under Developed Countries” OR “Under Developed Country” OR “under developed nations” OR “Under Developed World” OR “Under Served Population” OR “Under Served Populations” OR “Underdeveloped Countries” OR “Underdeveloped Country” OR “underdeveloped economies” OR “underdeveloped nations” OR “underdeveloped population” OR “Underdeveloped World” OR “Underserved Countries” OR “Underserved Nations” OR “Underserved Population” OR “Underserved Populations”		

	OR "Global South" OR Africa OR "Latin America" OR "South America" OR "Sub-Saharan Africa" OR Asia))		
Search string	#1 AND #2 AND #3 AND #4 AND #5	#6	6,080
Limits	Limit to English language; Limit to since year 2000; Limit document type (Article, Editorial)		
Total Result	#1 AND #2 AND #3 AND #4 AND #5 AND limits	#7	3,211

Web of Science (2021-05-10)

Concepts	Search strategy keywords	Search	# Results
Community-based programmes	ALL=(("community-based" OR "community based" OR community OR complex OR collective) AND (intervention OR program* OR trial OR evaluation OR action OR research))	#1	2,776,017
Social networks	ALL=("social network*" OR "social network analysis" OR "social network intervention*" OR "network analysis" OR "community networks" OR "social relationships" OR "social capital" OR "whole networks")	#2	134,632
Scale-up and sustainability	ALL=("scale-up" OR "scale up" OR sustain* OR implement* OR disseminat* OR "diffusion of innovation" OR "program evaluation")	#3	2,522,901
Non-communicable disease prevention (physical activity and diet)	ALL=(Physical activity OR exercise OR diet OR "non communicable" OR chronic OR health promotion OR weight)	#4	4,314,124
Low- and middle-income country setting	AB=((Afghanistan OR Albania OR Algeria OR "American Samoa" OR Angola OR Armenia OR Azerbaijan OR Bangladesh OR Belarus OR Byelarus OR Belorussia OR Belize OR Benin OR Bhutan OR Bolivia OR Bosnia OR Botswana OR Brazil OR Bulgaria OR Burma OR "Burkina Faso" OR Burundi OR "Cabo Verde" OR "Cape Verde" OR Cambodia OR Cameroon OR "Central African Republic" OR Chad OR China OR Colombia OR Comoros OR Comores OR Comoro OR Congo OR "Costa Rica" OR "Côte d'Ivoire" OR Cuba OR Djibouti OR Dominica OR "Dominican Republic" OR Ecuador OR Egypt OR "El Salvador" OR "Equatorial Guinea" OR Eritrea OR Ethiopia OR Fiji OR Gabon OR Gambia OR Gaza OR Georgia OR "Georgia Republic" OR Ghana OR Grenada OR Grenadines OR Guatemala OR Guinea OR Guinea- Bissau OR Guyana OR Haiti OR Herzegovina OR Hercegovina OR Honduras OR India OR Indonesia OR Iran OR Iraq OR "Ivory Coast" OR Jamaica OR	#5	2,096,577

	Jordan OR Kazakhstan OR Kenya OR Kiribati OR "Democratic People's Republic of Korea" OR Kosovo OR Kyrgyz OR Kirghizia OR Kirghiz OR Kyrgyzstan OR Lao PDR OR Laos OR Lebanon OR Lesotho OR Liberia OR Libya OR Macedonia OR Madagascar OR Malawi OR Malay OR Malaya OR Malaysia OR Maldives OR Mali OR "Marshall Islands" OR Mauritania OR Mauritius OR Mexico OR Micronesia OR Moldova OR Mongolia OR Montenegro OR Morocco OR Mozambique OR Myanmar OR Namibia OR Nepal OR Nicaragua OR Niger OR Nigeria OR Pakistan OR Palau OR "Papua New Guinea" OR Paraguay OR Peru OR Philippines OR Principe OR Romania OR Ruanda OR Rwanda OR Samoa OR "Sao Tome" OR Senegal OR Serbia OR "Sierra Leone" OR "Solomon Islands" OR Somalia OR "South Africa" OR "South Sudan" OR "Sri Lanka" OR "St Lucia" OR "St Vincent" OR Sudan OR Surinam OR Suriname OR Swaziland OR Syria OR "Syrian Arab Republic" OR Tajikistan OR Tadzhikistan OR Tajikistan OR Tadzhik OR Tanzania OR Thailand OR Timor OR Togo OR Tonga OR Tunisia OR Turkey OR Turkmen OR Turkmenistan OR Tuvalu OR Uganda OR Ukraine OR Uzbek OR Uzbekistan OR Vanuatu OR Venezuela OR Vietnam OR "West Bank" OR Yemen OR Zambia OR Zimbabwe) OR ("Deprived Countries" OR "Developing Countries" OR "Developing Country" OR "Developing Economies" OR "Developing Economy" OR "Developing Nation" OR "Developing Nations" OR "Developing World" OR "LAMI Countries" OR "LAMI Country" OR "Less Developed Countries" OR "Less Developed Country" OR "Less Developed Economies" OR "Less Developed Nation" OR "Less Developed Nations" OR "Less Developed World" OR "Lesser Developed Countries" OR "Lesser Developed Nations" OR LMIC OR LMICS OR "Low GDP" OR "Low GNP" OR "Low Gross Domestic" OR "Low Gross National" OR "Low Income" OR "Lower GDP" OR "lower gross domestic" OR "Lower Income" OR "Middle Income" OR "Poor Countries" OR "Poor Country" OR "Poor Economies" OR "Poor Economy" OR "Poor Nation" OR "Poor Nations" OR "Poor Population" OR "Poor Populations" OR "poor world" OR "Poorer Countries" OR "Poorer Economies" OR "Poorer Economy" OR "Poorer Nations" OR "Poorer Population" OR "Poorer Populations" OR "Third World" OR "Transitional Countries" OR "Transitional Country" OR "Transitional Economies" OR "Transitional Economy" OR "Under Developed Countries" OR "Under Developed Country" OR "under developed nations" OR "Under Developed World" OR "Under Served Population" OR "Under Served Populations" OR "Underdeveloped Countries" OR "Underdeveloped Country" OR "underdeveloped economies" OR "underdeveloped nations" OR "underdeveloped		

	population” OR “Underdeveloped World” OR “Underserved Countries” OR “Underserved Nations” OR “Underserved Population” OR “Underserved Populations” OR “Global South” OR Africa OR “Latin America” OR “South America” OR “Sub-Saharan Africa” OR Asia))		
Search string	#1 AND #2 AND #3 AND #4 AND #5	#6	172
Limits	Limit to English language; Limit to since year 2000		
Total Result	#1 AND #2 AND #3 AND #4 AND #5 AND limits	#7	162

CINAHL-EBSCO (2021-05-10)

Concepts	Search strategy keywords	Search	# Results
Community-based programmes	TX ("community-based" OR "community based" OR community OR complex OR collective) AND (intervention OR program* OR trial OR evaluation OR action OR research)	#1	480,307
Social networks	TX ("social network*" OR "social network analysis" OR "social network intervention*" OR "network analysis" OR "community networks" OR “social relationships” OR “social capital” OR “whole networks”)	#2	37,252
Scale-up and sustainability	TX ("scale-up" OR "scale up" OR sustain* OR implement* OR disseminat* OR "diffusion of innovation" OR "program evaluation")	#3	388,571
Non-communicable disease prevention (physical activity and diet)	TX (Physical activity OR exercise OR diet OR "non communicable" OR chronic OR health promotion OR weight)	#4	1,025,075
Low- and middle-income country setting	TX ((Afghanistan OR Albania OR Algeria OR “American Samoa” OR Angola OR Armenia OR Azerbaijan OR Bangladesh OR Belarus OR Byelarus OR Belorussia OR Belize OR Benin OR Bhutan OR Bolivia OR Bosnia OR Botswana OR Brazil OR Bulgaria OR Burma OR “Burkina Faso” OR Burundi OR “Cabo Verde” OR “Cape Verde” OR Cambodia OR Cameroon OR “Central African Republic” OR Chad OR China OR Colombia OR Comoros OR Comores OR Comoro OR Congo OR “Costa Rica” OR “Côte d'Ivoire” OR Cuba OR Djibouti OR Dominica OR “Dominican Republic” OR Ecuador OR Egypt OR “El Salvador” OR “Equatorial Guinea” OR Eritrea OR Ethiopia OR Fiji OR Gabon OR Gambia OR Gaza OR Georgia OR “Georgia Republic” OR Ghana OR Grenada OR Grenadines OR Guatemala OR Guinea OR Guinea- Bissau OR Guyana OR Haiti OR Herzegovina OR Hercegovina OR	#5	1,199,237

	Honduras OR India OR Indonesia OR Iran OR Iraq OR "Ivory Coast" OR Jamaica OR Jordan OR Kazakhstan OR Kenya OR Kiribati OR "Democratic People's Republic of Korea" OR Kosovo OR Kyrgyz OR Kirghizia OR Kirghiz OR Kyrgyzstan OR Lao PDR OR Laos OR Lebanon OR Lesotho OR Liberia OR Libya OR Macedonia OR Madagascar OR Malawi OR Malay OR Malaya OR Malaysia OR Maldives OR Mali OR "Marshall Islands" OR Mauritania OR Mauritius OR Mexico OR Micronesia OR Moldova OR Mongolia OR Montenegro OR Morocco OR Mozambique OR Myanmar OR Namibia OR Nepal OR Nicaragua OR Niger OR Nigeria OR Pakistan OR Palau OR "Papua New Guinea" OR Paraguay OR Peru OR Philippines OR Principe OR Romania OR Ruanda OR Rwanda OR Samoa OR "Sao Tome" OR Senegal OR Serbia OR "Sierra Leone" OR "Solomon Islands" OR Somalia OR "South Africa" OR "South Sudan" OR "Sri Lanka" OR "St Lucia" OR "St Vincent" OR Sudan OR Surinam OR Suriname OR Swaziland OR Syria OR "Syrian Arab Republic" OR Tajikistan OR Tadzhikistan OR Tajikistan OR Tadzhik OR Tanzania OR Thailand OR Timor OR Togo OR Tonga OR Tunisia OR Turkey OR Turkmen OR Turkmenistan OR Tuvalu OR Uganda OR Ukraine OR Uzbek OR Uzbekistan OR Vanuatu OR Venezuela OR Vietnam OR "West Bank" OR Yemen OR Zambia OR Zimbabwe) OR ("Deprived Countries" OR "Developing Countries" OR "Developing Country" OR "Developing Economies" OR "Developing Economy" OR "Developing Nation" OR "Developing Nations" OR "Developing World" OR "LAMI Countries" OR "LAMI Country" OR "Less Developed Countries" OR "Less Developed Country" OR "Less Developed Economies" OR "Less Developed Nation" OR "Less Developed Nations" OR "Less Developed World" OR "Lesser Developed Countries" OR "Lesser Developed Nations" OR LMIC OR LMICS OR "Low GDP" OR "Low GNP" OR "Low Gross Domestic" OR "Low Gross National" OR "Low Income" OR "Lower GDP" OR "lower gross domestic" OR "Lower Income" OR "Middle Income" OR "Poor Countries" OR "Poor Country" OR "Poor Economies" OR "Poor Economy" OR "Poor Nation" OR "Poor Nations" OR "Poor Population" OR "Poor Populations" OR "poor world" OR "Poorer Countries" OR "Poorer Economies" OR "Poorer Economy" OR "Poorer Nations" OR "Poorer Population" OR "Poorer Populations" OR "Third World" OR "Transitional Countries" OR "Transitional Country" OR "Transitional Economies" OR "Transitional Economy" OR "Under Developed Countries" OR "Under Developed Country" OR "under developed nations" OR "Under Developed World" OR "Under Served Population" OR "Under Served Populations" OR "Underdeveloped Countries" OR "Underdeveloped Country" OR		

	"underdeveloped economies" OR "underdeveloped nations" OR "underdeveloped population" OR "Underdeveloped World" OR "Underserved Countries" OR "Underserved Nations" OR "Underserved Population" OR "Underserved Populations" OR "Global South" OR Africa OR "Latin America" OR "South America" OR "Sub-Saharan Africa" OR Asia))		
Search string	#1 AND #2 AND #3 AND #4 AND #5	#6	274
Limits	Limit to English language; Limit to since year 2000		
Total Result	#1 AND #2 AND #3 AND #4 AND #5 AND limits	#7	258

SocINDEX (2021-05-10)

**Concepts**	**Search strategy keywords**	**Search**	**# Results**
Community-based programmes	("community-based" OR "community based" OR community OR complex OR collective) AND (intervention OR program* OR trial OR evaluation OR action OR research)	#1	240,457
Social networks	("social network*" OR "social network analysis" OR "social network intervention*" OR "network analysis" OR "community networks" OR "social relationships" OR "social capital" OR "whole networks")	#2	80,615
Scale-up and sustainability	("scale-up" OR "scale up" OR sustain* OR implement* OR disseminat* OR "diffusion of innovation" OR "program evaluation")	#3	132,008
Non-communicable disease prevention (physical activity and diet)	(Physical activity OR exercise OR diet OR "non communicable" OR chronic OR health promotion OR weight)	#4	76,756
Low- and middle-income country setting	((Afghanistan OR Albania OR Algeria OR "American Samoa" OR Angola OR Armenia OR Azerbaijan OR Bangladesh OR Belarus OR Byelarus OR Belorussia OR Belize OR Benin OR Bhutan OR Bolivia OR Bosnia OR Botswana OR Brazil OR Bulgaria OR Burma OR "Burkina Faso" OR Burundi OR "Cabo Verde" OR "Cape Verde" OR Cambodia OR Cameroon OR "Central African Republic" OR Chad OR China OR Colombia OR Comoros OR Comores OR Comoro OR Congo OR "Costa Rica" OR "Côte d'Ivoire" OR Cuba OR Djibouti OR Dominica OR "Dominican Republic" OR Ecuador OR Egypt OR "El Salvador" OR "Equatorial Guinea" OR Eritrea OR Ethiopia OR Fiji OR Gabon OR Gambia OR Gaza OR Georgia OR "Georgia Republic" OR Ghana OR Grenada OR Grenadines OR Guatemala OR Guinea OR Guinea-	#5	428,329

	Bissau OR Guyana OR Haiti OR Herzegovina OR Hercegovina OR Honduras OR India OR Indonesia OR Iran OR Iraq OR “Ivory Coast” OR Jamaica OR Jordan OR Kazakhstan OR Kenya OR Kiribati OR “Democratic People’s Republic of Korea” OR Kosovo OR Kyrgyz OR Kirghizia OR Kirghiz OR Kyrgyzstan OR Lao PDR OR Laos OR Lebanon OR Lesotho OR Liberia OR Libya OR Macedonia OR Madagascar OR Malawi OR Malay OR Malaya OR Malaysia OR Maldives OR Mali OR “Marshall Islands” OR Mauritania OR Mauritius OR Mexico OR Micronesia OR Moldova OR Mongolia OR Montenegro OR Morocco OR Mozambique OR Myanmar OR Namibia OR Nepal OR Nicaragua OR Niger OR Nigeria OR Pakistan OR Palau OR “Papua New Guinea” OR Paraguay OR Peru OR Philippines OR Principe OR Romania OR Ruanda OR Rwanda OR Samoa OR “Sao Tome” OR Senegal OR Serbia OR “Sierra Leone” OR “Solomon Islands” OR Somalia OR “South Africa” OR “South Sudan” OR “Sri Lanka” OR “St Lucia” OR “St Vincent” OR Sudan OR Surinam OR Suriname OR Swaziland OR Syria OR “Syrian Arab Republic” OR Tajikistan OR Tadzhikistan OR Tajikistan OR Tadzhik OR Tanzania OR Thailand OR Timor OR Togo OR Tonga OR Tunisia OR Turkey OR Turkmen OR Turkmenistan OR Tuvalu OR Uganda OR Ukraine OR Uzbek OR Uzbekistan OR Vanuatu OR Venezuela OR Vietnam OR “West Bank” OR Yemen OR Zambia OR Zimbabwe) OR (“Deprived Countries” OR “Developing Countries” OR “Developing Country” OR “Developing Economies” OR “Developing Economy” OR “Developing Nation” OR “Developing Nations” OR “Developing World” OR “LAMI Countries” OR “LAMI Country” OR “Less Developed Countries” OR “Less Developed Country” OR “Less Developed Economies” OR “Less Developed Nation” OR “Less Developed Nations” OR “Less Developed World” OR “Lesser Developed Countries” OR “Lesser Developed Nations” OR LMIC OR LMICS OR “Low GDP” OR “Low GNP” OR “Low Gross Domestic” OR “Low Gross National” OR “Low Income” OR “Lower GDP” OR “lower gross domestic” OR “Lower Income” OR “Middle Income” OR “Poor Countries” OR “Poor Country” OR “Poor Economies” OR “Poor Economy” OR “Poor Nation” OR “Poor Nations” OR “Poor Population” OR “Poor Populations” OR “poor world” OR “Poorer Countries” OR “Poorer Economies” OR “Poorer Economy” OR “Poorer Nations” OR “Poorer Population” OR “Poorer Populations” OR “Third World” OR “Transitional Countries” OR “Transitional Country” OR “Transitional Economies” OR “Transitional Economy” OR “Under Developed Countries” OR “Under Developed Country” OR “under developed nations” OR “Under Developed World” OR “Under Served Population” OR “Under Served Populations” OR “Underdeveloped Countries” OR “Underdeveloped		

	Country" OR "underdeveloped economies" OR "underdeveloped nations" OR "underdeveloped population" OR "Underdeveloped World" OR "Underserved Countries" OR "Underserved Nations" OR "Underserved Population" OR "Underserved Populations" OR "Global South" OR Africa OR "Latin America" OR "South America" OR "Sub-Saharan Africa" OR Asia))		
Search string	#1 AND #2 AND #3 AND #4 AND #5	#6	32
Limits	Limit to English language; Limit to since year 2000		
Total Result	#1 AND #2 AND #3 AND #4 AND #5 AND limits	#7	29

Cochrane (2021-05-10)

**Concepts**	**Search strategy keywords**	**Search**	**# Results**
Community-based programmes	("community-based" OR "community based" OR community OR complex OR collective) AND (intervention OR program* OR trial OR evaluation OR action OR research)<br>AND MeSH descriptor: [Community-Based Participatory Research] explode all trees	#1	86,794
Social networks	("social network*" OR "social network analysis" OR "social network intervention*" OR "network analysis" OR "community networks" OR "social relationships" OR "social capital" OR "whole networks")<br>AND MeSH descriptor: [Social Network Analysis] explode all trees	#2	1,514
Scale-up and sustainability	("scale-up" OR "scale up" OR sustain* OR implement* OR disseminat* OR "diffusion of innovation" OR "program evaluation")<br>AND MeSH descriptor: [Program Evaluation] explode all trees	#3	105,571
Non-communicable disease prevention (physical activity and diet)	(Physical activity OR exercise OR diet OR "non communicable" OR chronic OR health promotion OR weight)<br>AND MeSH descriptor: [Noncommunicable Diseases] explode all trees	#4	326,399
Low- and middle-income country setting	Not able to complete on this database		–
Search string	#1 AND #2 AND #3 AND #4	#5	304
Limits	Limit to exclude protocols; Limit to since year 2000		
Total Result	#1 AND #2 AND #3 AND #4 AND limits	#6	268

International Bibliography of the Social Sciences (2021-05-10)

Concepts	Search strategy keywords	Search	# Results
Community-based programmes	Ab(("community-based" OR "community based" OR community OR complex OR collective) AND (intervention OR program* OR trial OR evaluation OR action OR research))	#1	108,621
Social networks	Ab("social network*" OR "social network analysis" OR "social network intervention*" OR "network analysis" OR "community networks" OR “social relationships” OR “social capital” OR “whole networks”)	#2	29,987
Scale-up and sustainability	Ab("scale-up" OR "scale up" OR sustain* OR implement* OR disseminat* OR "diffusion of innovation" OR "program evaluation")	#3	186,317
Non-communicable disease prevention (physical activity and diet)	Anywhere (Physical activity OR exercise OR diet OR "non communicable" OR chronic OR health promotion OR weight)	#4	378,734
Low- and middle-income country setting	Anywhere ((Afghanistan OR Albania OR Algeria OR “American Samoa” OR Angola OR Armenia OR Azerbaijan OR Bangladesh OR Belarus OR Byelarus OR Belorussia OR Belize OR Benin OR Bhutan OR Bolivia OR Bosnia OR Botswana OR Brazil OR Bulgaria OR Burma OR “Burkina Faso” OR Burundi OR “Cabo Verde” OR “Cape Verde” OR Cambodia OR Cameroon OR “Central African Republic” OR Chad OR China OR Colombia OR Comoros OR Comores OR Comoro OR Congo OR “Costa Rica” OR “Côte d'Ivoire” OR Cuba OR Djibouti OR Dominica OR “Dominican Republic” OR Ecuador OR Egypt OR “El Salvador” OR “Equatorial Guinea” OR Eritrea OR Ethiopia OR Fiji OR Gabon OR Gambia OR Gaza OR Georgia OR “Georgia Republic” OR Ghana OR Grenada OR Grenadines OR Guatemala OR Guinea OR Guinea- Bissau OR Guyana OR Haiti OR Herzegovina OR Hercegovina OR Honduras OR India OR Indonesia OR Iran OR Iraq OR “Ivory Coast” OR Jamaica OR Jordan OR Kazakhstan OR Kenya OR Kiribati OR “Democratic People’s Republic of Korea” OR Kosovo OR Kyrgyz OR Kirghizia OR Kirghiz OR Kyrgyzstan OR Lao PDR OR Laos OR Lebanon OR Lesotho OR Liberia OR Libya OR Macedonia OR Madagascar OR Malawi OR Malay OR Malaya OR Malaysia OR Maldives OR Mali OR “Marshall Islands” OR Mauritania OR Mauritius OR Mexico OR Micronesia OR Moldova OR Mongolia OR Montenegro OR Morocco OR Mozambique OR Myanmar OR Namibia OR Nepal OR Nicaragua OR Niger OR Nigeria OR Pakistan OR Palau OR “Papua New Guinea” OR	#5	1,602,554

	Paraguay OR Peru OR Philippines OR Principe OR Romania OR Ruanda OR Rwanda OR Samoa OR “Sao Tome” OR Senegal OR Serbia OR “Sierra Leone” OR “Solomon Islands” OR Somalia OR “South Africa” OR “South Sudan” OR “Sri Lanka” OR “St Lucia” OR “St Vincent” OR Sudan OR Surinam OR Suriname OR Swaziland OR Syria OR “Syrian Arab Republic” OR Tajikistan OR Tadzhikistan OR Tajikistan OR Tadzhik OR Tanzania OR Thailand OR Timor OR Togo OR Tonga OR Tunisia OR Turkey OR Turkmen OR Turkmenistan OR Tuvalu OR Uganda OR Ukraine OR Uzbek OR Uzbekistan OR Vanuatu OR Venezuela OR Vietnam OR “West Bank” OR Yemen OR Zambia OR Zimbabwe) OR (“Deprived Countries” OR “Developing Countries” OR “Developing Country” OR “Developing Economies” OR “Developing Economy” OR “Developing Nation” OR “Developing Nations” OR “Developing World” OR “LAMI Countries” OR “LAMI Country” OR “Less Developed Countries” OR “Less Developed Country” OR “Less Developed Economies” OR “Less Developed Nation” OR “Less Developed Nations” OR “Less Developed World” OR “Lesser Developed Countries” OR “Lesser Developed Nations” OR LMIC OR LMICS OR “Low GDP” OR “Low GNP” OR “Low Gross Domestic” OR “Low Gross National” OR “Low Income” OR “Lower GDP” OR “lower gross domestic” OR “Lower Income” OR “Middle Income” OR “Poor Countries” OR “Poor Country” OR “Poor Economies” OR “Poor Economy” OR “Poor Nation” OR “Poor Nations” OR “Poor Population” OR “Poor Populations” OR “poor world” OR “Poorer Countries” OR “Poorer Economies” OR “Poorer Economy” OR “Poorer Nations” OR “Poorer Population” OR “Poorer Populations” OR “Third World” OR “Transitional Countries” OR “Transitional Country” OR “Transitional Economies” OR “Transitional Economy” OR “Under Developed Countries” OR “Under Developed Country” OR “under developed nations” OR “Under Developed World” OR “Under Served Population” OR “Under Served Populations” OR “Underdeveloped Countries” OR “Underdeveloped Country” OR “underdeveloped economies” OR “underdeveloped nations” OR “underdeveloped population” OR “Underdeveloped World” OR “Underserved Countries” OR “Underserved Nations” OR “Underserved Population” OR “Underserved Populations” OR “Global South” OR Africa OR “Latin America” OR “South America” OR “Sub-Saharan Africa” OR Asia))		
Search string	#1 AND #2 AND #3 AND #4 AND #5	#6	2,727
Limits	Limit to English language; Limit to since year 2000; Limit to exclude ("Dissertations & Theses" OR "Conference Papers & Proceedings" OR "Books" OR "Working Papers")		

Total Result	#1 AND #2 AND #3 AND #4 AND #5 AND limits	#7	1,590

Journal and Google basic search (2021-05-10)

**Concepts**	**Search strategy keywords**	**Search**	**# Results**
Social Networks	("scale-up" OR "sustainability" OR "implementation") AND ("physical activity" OR "diet" OR "chronic" OR "non-communicable" OR health promotion OR weight), since 2000, excl. books	#1	246
Applied Network Science	("scale-up" OR "sustainability" OR "implementation") AND ("physical activity" OR "diet" OR "chronic" OR "non-communicable" OR health promotion OR weight), since 2000, excl. books	#1	31
Journal of Social Structure	("scale-up" OR "sustainability" OR "implementation") AND ("physical activity" OR "diet" OR "chronic" OR "non-communicable" OR health promotion OR weight), since 2000, excl. books	#1	3
Google Scholar (UK based)	(“social network” OR “network analysis” OR “social capital” OR “social relationships” AND ("scale up" OR sustain OR implement) AND ("physical activity" OR diet OR chronic OR "non communicable") AND ("low income" OR "middle income" OR LMIC), since 2000	First 3 pages	30
Google (UK based)	(“social network” OR “network analysis” OR “social capital” OR “social relationships”) AND ("scale up" OR sustain OR implement) AND ("physical activity" OR diet OR chronic OR "non communicable") AND ("low income" OR "middle income" OR LMIC), since 2000	First 3 pages	30
