# Supplementary material for: Using social networks to scale up and sustain community-based programmes to improve physical activity and diet in low-income and middle-income countries: a scoping review protocol
Source: BMJ Open. 2021 Sep 14;11(9):e053586. doi: 10.1136/bmjopen-2021-053586 (PMC8442048; doi:10.1136/bmjopen-2021-053586)
Supplement: Author's manuscript [file bmjopen-2021-053586.draft_revisions.pdf]

BMJ Open is committed to open peer review. As part of this commitment we make the peer review history of every article we publish publicly available.

When an article is published we post the peer reviewers' comments and the authors' responses online. We also post the versions of the paper that were used during peer review. These are the versions that the peer review comments apply to.

The versions of the paper that follow are the versions that were submitted during the peer review process. They are not the versions of record or the final published versions. They should not be cited or distributed as the published version of this manuscript.

BMJ Open is an open access journal and the full, final, typeset and author-corrected version of record of the manuscript is available on our site with no access controls, subscription charges or pay-per-view fees (http://bmjopen.bmj.com).

If you have any questions on BMJ Open's open peer review process please email info.bmjopen@bmj.com

BMJ Open

Using social networks to scale-up and sustain community-based programmes in low- and middle-income countries: a systematic scoping review protocol.

Journal:	*BMJ Open*
Manuscript ID	bmjopen-2021-053586
Article Type:	Protocol
Date Submitted by the Author:	27-May-2021

Complete List of Authors:	Abrahams, Nina; University of Bristol, Exercise, Nutrition, and Health Sciences; Faculty of Health Sciences University of Cape Town, Health through Physical Activity Lifestyle and Sport Research Centre<br>Lambert, Estelle; University of Cape Town, Health through Physical Activity Lifestyle and Sport Research Centre<br>Marais, Frederick; Western Cape Government, Department of Health ; North-West University, Africa Unit for Transdisciplinary Health Research<br>Toumpakari, Zoi; University of Bristol, Centre for Exercise, Nutrition, and Health Sciences<br>Foster, Charlie ; University of Bristol, Centre for Exercise, Nutrition, and Health Sciences

Keywords:	PUBLIC HEALTH, Quality in health care < HEALTH SERVICES ADMINISTRATION & MANAGEMENT, QUALITATIVE RESEARCH

SCHOLARONE™ Manuscripts

I, the Submitting Author has the right to grant and does grant on behalf of all authors of the Work (as defined in the below author licence), an exclusive licence and/or a non-exclusive licence for contributions from authors who are: i) UK Crown employees; ii) where BMJ has agreed a CC-BY licence shall apply, and/or iii) in accordance with the terms applicable for US Federal Government officers or employees acting as part of their official duties; on a worldwide, perpetual, irrevocable, royalty-free basis to BMJ Publishing Group Ltd ("BMJ") its licensees and where the relevant Journal is co-owned by BMJ to the co-owners of the Journal, to publish the Work in this journal and any other BMJ products and to exploit all rights, as set out in our licence.

The Submitting Author accepts and understands that any supply made under these terms is made by BMJ to the Submitting Author unless you are acting as an employee on behalf of your employer or a postgraduate student of an affiliated institution which is paying any applicable article publishing charge ("APC") for Open Access articles. Where the Submitting Author wishes to make the Work available on an Open Access basis (and intends to pay the relevant APC), the terms of reuse of such Open Access shall be governed by a Creative Commons licence – details of these licences and which Creative Commons licence will apply to this Work are set out in our licence referred to above.

Other than as permitted in any relevant BMJ Author's Self Archiving Policies, I confirm this Work has not been accepted for publication elsewhere, is not being considered for publication elsewhere and does not duplicate material already published. I confirm all authors consent to publication of this Work and authorise the granting of this licence.

For peer review only

Using social networks to scale-up and sustain community-based programmes in low- and middle-income countries: a systematic scoping review protocol.

Nina Abrahams[*1,2], Estelle V. Lambert[2], Frederick Marais[3,4], Zoi Toumpakari[1], Charlie Foster[1]

[1] Centre for Exercise, Nutrition and Health Sciences, School for Policy Studies, Faculty of Social Sciences and Law, University of Bristol, Bristol, United Kingdom
[2] Health through Physical Activity Lifestyle and Sport Research Centre, Lifestyle and Sport, Division of Exercise Science and Sports Medicine, Department of Human Biology, Faculty of Health Sciences, University of Cape Town, Cape Town, South Africa
[3] Increasing Wellness, Department of Health, Western Cape Government, South Africa
[4] Africa Unit for Transdisciplinary Health Research, North West University, South Africa

[*]Correspondence to Nina Abrahams; ntabrahams@gmail.com

For peer review only

Abstract

Introduction The World Health Organisation endorses community-based programmes as a cost-effective, feasible, and a 'best buy' in the prevention and management of non-communicable diseases. These programmes are particularly successful when the community actively participates in its design, implementation, and evaluation. However, they may be only useful insofar as they can be scaled-up and sustained in some meaningful way. Social network research may serve as an important tool for determining the underlying mechanisms that contribute to this process. The aim of this planned scoping review is to map and collate literature on the role of social networks in scaling-up and sustaining community-based programmes in low- and middle-income countries. The findings will be used to determine the scope of research, identify gaps in the literature, and contribute to a broader research investigation.

Methods and analysis This protocol has been planned around the Arksey and O'Malley framework and its enhancement. The search strategy will cover terms related to the three main components in this study: social networks, community-based programmes, and scale-up/sustainability – within non-communicable disease research. The following databases will be used for this review: PubMed, Cochrane, Scopus, Web of Science, CINAHL, SocIndex, the International Bibliography of the Social Sciences, and Google Scholar. Studies must have been published since 2000 and based in a low- or middle-income country. Books, conference abstracts, and research focused only on children will be excluded. Two reviewers will screen and analyse the publications. Included publications will be thematically analysed using the Framework Approach.

Ethics and dissemination Ethical approval will not be sought for this review as no individual-level data or human participants will be involved. The findings from the review will be published in an accredited journal. The PRISMA-ScR checklist will be used to support transparency and guide translation of the review.

Strengths and limitations of this study
➢ This protocol will be used to explore the role of social networks in the under-researched area of scaling-up and sustainaining community-based programmes in low- and middle-income countries.
➢ This protocol is based on a widely-used framework for scoping reviews and will make use of iterative steps to continosuly improve rigour.
➢ The protocol findings will contribute to a broader research project.
➢ Although comprehensive, the protocol has limitations with regards to search terms, language, and databases used.
➢ Quality of evidence will not be evaluated in this scoping review.

For peer review only

Introduction

The World Health Organisation endorses a set of 16 programmes that are shown to be cost-effective, feasible, and so 'best buys' in the prevention and management of non-communicable diseases [NCDs] at an individual and population level.(1) One of the 'best buy' programmes for both physical activity and diet are community-based awareness, educational, and behaviour change programmes. These community-based programmes [CBPs] vary in practice and so do not have a set definition but refer broadly to programmes that target and engage a defined population in activities ranging widely from group-based interventions or mass media campaigns to environmental, structural or policy changes that are adapted to, set in, and ideally delivered by the community for that community.(2-8) Programmes wherein communities actively participate and lead in design, implementation, and evaluation have been shown to be particularly creative, sustainable, and effective in improving health outcomes and developing longer-term confidence and capacity building within communities.(7, 9, 10) These programmes are not clinical or pharmaceutical-based but are more focused on awareness, education, and creating a supportive environment for behaviour change, social change, and community development.(1, 2, 10)

One could argue that CBPs are only truly useful insofar as they can be scaled-up and sustained in some meaningful way. If the intervention does not reach enough people, then their effects are spread thin and the CBP has less chance of making a sustainable and significant impact.(2, 3, 5, 11-13) Scale-up of an intervention has been defined as *"the process by which health interventions shown to be efficacious on a small scale and/or under controlled conditions are expanded under real world conditions into broader policy or practice."*(14) This can refer to the intervention or programme being introduced to a greater number of its target population, being adapted for other populations, or becoming increasingly ratified at a policy and environmental level which may then lead to greater

impacts on a wider population.(12, 14-16) Sustainability of health programmes has been defined as *"the program continues to be delivered and/or individual behaviour change is maintained; the program and individual behaviour change may evolve or adapt while continuing to produce benefits for individuals/systems, after a defined period of time."*(17)

There is, however, limited research on how to effectively scale-up and sustain complex community-based programmes.(14, 15) This dearth of literature is particularly pertinent in low- and middle-income countries [LMICs]. For example, in 2016, Reis et al. [18] conducted a systematic review on scaling-up physical activity interventions. The authors only identified 16 scaled-up interventions in peer-reviewed literature, of which only two were based in an LMIC.

One potentially useful tool for improving the theory and practice of the scale-up and sustainability of CBPs is social network research [SNR]. SNR is research that examines the relationship between actors (individuals or organisations) in a system.(18, 19) The aim of SNR is to identify who is in a particular network, what their attributes are, and who they are connected to in the network and then to visually plot these relationship on a network graph.(19) There are various ways in which network structures can then be analysed.(20-22) For example, one can calculate the density of the network (how many ties/relationships there are between actors) where a high degree of ties to one stakeholder may indicate that they are considered a leader. Alternatively, there may be several densely tied sub-groups within a bigger network with bridging actors who connect these clusters. Once one understands how the network is functioning, one can then try and utilise these connections by generating strategies for greater and more efficient network reach and sustainability.(19, 23, 24) Valente (23) suggests four main 'network interventions': identifying key actors, identifying and shifting the actions of sub-clusters at a time, stimulating peer-to-peer influence, and altering the network (removing or adding actors into key network positions). In 2019, Hunter et al. (25) conducted a systematic review and meta-analysis of randomised controlled trials to identify the effectiveness of social network interventions over a range of health behaviours and outcomes. The pooled evidence indicates that social network interventions result in improved health outcomes and are particularly useful at reaching and retaining underserved populations. However, of the 37 included interventions only six studies were based in a LMIC, none of which took place in Africa.

To fill this gap, the authors (NTA, CF, EVL, FM, ZT) are conducting research to understand the various attributes of social networks that may impact or influence the successful implementation, sustainability and scale-up of community-based, NCD prevention, programmes. The aim of this planned scoping review is therefore to map and collate literature on the role of social networks for scaling-up and sustaining NCD prevention CBPs in LMICs. The findings of this review will be used to

For peer review only

determine the current scope of research, help to identify gaps in the literature, and support the development of a programme theory as part of the broader research project aims.

Scoping review research questions

The overarching research question is: *Is there research on social networks within scale-up studies of community-based programmes in low- and middle-income countries? And if so, what is the nature of that role, and the characteristics of the social networks in providing that function?*

The sub-questions that will guide the scoping review are presented in table 1.

Table 1. Scoping review questions	
**Descriptive**	What is the volume of publications?
	What are the research designs of the publications?
	What is the geographical scope of the publications?
	What settings are used for the programmes?
**Social networks**	What types of networks and/or network interventions are described in the publications?
	Who is involved in the network(s)?
	What value, if any, do social networks bring to community-based programmes?
**Community-based programmes**	What types of community-based programmes are covered?
	What activities are included in the community-based programmes?
	What theories/theoretical approaches underpin the community-based programmes?
**Scale-up and sustainability**	What scale-up/sustainability theories are utilised in the publications?
	How is scale-up and/or sustainability conceptualised or operationalised?
**Mechanisms**	Are any potential mechanisms of scale-up and sustainability explored in the publications?

Methods and analysis

Scoping reviews are useful for determining the broad scale and range of a body of literature and exploring key factors related to a particular concept of interest.(26, 27) This is particularly useful when there is a paucity of evidence in that field.(27) Scoping reviews are not intended for answering a specific question in detail but rather to gain a broad understanding of a particular topic.

This protocol has been planned around the methodological framework for scoping reviews outlined by the Arksey and O'Malley framework (28) as well as its enhancement.(27) The method and final reporting will also be based on the checklist provided by the Preferred Reporting Items for Systematic Reviews and Meta-analyses extension for Scoping Reviews [PRISMA-ScR].(29) These frameworks acknowledge that scoping reviews are an iterative process and that continuous

For peer review only

engagement and refinement is needed to retrieve relevant publications and analyse reliably.(27) Any iterations or deviations from this protocol will be reported in the final findings' publication.

Conceptual framework

This planned scoping review will form part of a larger research project that is framed by a realist evaluation. Realist evaluation is a theory-based evaluation strategy that aims to answer the question 'what works, how or why does this work, for whom, and in what circumstances?'.(30-33) Namely, a theory about how a programme is thought to operate is generated based on known literature, this initial theory is then tested on a real-world case study and, based on the results, the programme theory is refined. The programme theory is built using the structure: resource mechanisms-context-reasoning mechanisms-outcomes. Otherwise denoted as 'M(Resources)+C→M(Reasoning)=O' or [MCMO].(33)

In this research, the resource mechanism will be the social network structure of a public health intervention, such as, who the actors are, how many sub-groups there are in the network, and whether any network interventions have been used to alter the network. The context is the intervention itself and the social and geographical environment within which it operates. The outcome will be increased scale-up and sustainability. Variables are defined as:

1. Community based programmes
   - CBPs include any NCD prevention programmes or interventions that aim to change a defined population's psychological thinking, social interactions, or behaviours. They exclude interventions on a clinical or pharmaceutical level.
     - A broad definition of NCD prevention will be used to include any programme that promotes health and prevents non-communicable diseases.(34) Activities can include, but are not limited to, promotion of physical activity, healthy diets, or reducing sendentary behaviour.
     - CBPs may include programmes that use a defined community as a setting or programmes that include the community in the design, running, and evaluation of the intervention.(7)
     - Activities and outcomes of CBPs may include, but are not limited to, mass media education and awareness campaigns, group-based education or activity classes, individual motivational interviewing or brief behaviour change counselling, changing built environments, improving access to resources, community capacity building and empowerment, and improved social cohesion.(2, 9, 10, 35)

For peer review only

2. Scale-up and/or sustainability
   - Scale-up must be emphasised by the authors as an outcome or variable of interest. It can be horizontal (different populations or more of the population) or vertical (embedded in policy) scale-up.(15)
   - Sustainability must be emphasised by the authors as an outcome or variable of interest. It can include any interest in the programme and its effects continuing after initial implementation.(17)
3. Social networks
   - A broad definition of social networks will be used in line with previous systematic reviews of social network interventions.(25) Social networks encompass any social interaction or relationship between people or organisations, in vivo or online.
   - To be included a publication must have measured or considered these relationships in some aspect of the intervention design or delivery. This includes recognising an existing social network as being important to the success of the intervention (social network) or actively manipulating a network as part of the scale-up or implementation strategy (social network intervention).
4. Actors
   - Any of the people or organisations mentioned that play a role in the CBP (such as intervention developers, implementers, community members, participants, funders, policy makers).
5. Reasoning mechanisms
   - Underlying social or psychological responses that potentially foster or inhibit the outcome of scale-up and/or sustainability (such as trust or communication patterns).
6. Low- and middle-income countries
   - Countries that are low-income, lower-middle income, or upper-middle income economies as categorised by the World Bank in 2021.(36)

Inclusion and exclusion criteria

To be included, articles must explore network, context, and outcome components. Namely they must explore the role that the structure of a social network may have to influence scaling-up and/or sustaining community-based programmes. At least two of these components should be highlighted in the abstract or summary of the publications to be considered for further review. Included articles can be peer-reviewed frameworks, theoretical papers, reviews, or primary (quantitative, qualitative, or mixed method) studies or grey literature, published in English since 2000. This start date is based

For peer review only

on the rapid rise of CBP and scale-up literature since 2000 on the International Bibliography of the Social Sciences [IBSS] database. The cut-off date will be reflected in the final scoping review report. Inclusion population are human adults (19-64) and older adults (>65). If the publication has a wide age range, then the mean age should be >18 or more than 50% of the participants or beneficiaries should fall within the age range. Articles will be included if they have a wide age range but have analysed adult and child populations separately. Child or school-based only programmes are not included in the review at this stage as the broader research project focuses on adult population networks. In addition, only publications that include low- and middle-income contexts or considerations in their analysis will be considered. Excluded studies include conference abstracts, protocols, books, any outputs not published in English, and publications only focused on high-income countries (Table 2).

Considering the expected dearth of literature, the proposed scoping review will be kept broad. However, as this review follows an iterative process, inclusion and exclusion criteria may be further refined after the initial retrieval and review of abstracts to closer reflect the larger research project aims.(27) For example, CBPs may be further refined to only include multi-component (complex) programmes. Namely, programmes with more than one activity or component.(3, 31) In addition, articles may be refined to only include CBPs wherein the community has been intimately involved in developing and implementing the intervention.(7, 9) Focus may also be given specifically to physical activity and nutrition programmes. Any adaptions to the inclusion criteria will be reported in the final publication.

Table 2. Inclusion and exclusion criteria

**Inclusion criteria**	**Exclusion criteria**
Articles exploring the role of social networks in the scale-up and/or sustainability of NCD prevention community-based programmes	Books, book chapters, book reviews
Peer-reviewed articles, including original research, reviews, commentaries, and opinion pieces	Conference proceedings, dissertations/theses, and abstracts
Grey literature (e.g., institution reports, government documents)	Protocols
Indexed in PubMed, CINAHL, Scopus, Web of Science, Cochrane, IBSS, Google (Scholar)	Website, newspaper, and social media content
Published from year 2000 onwards	Published before 01 Jan 2000
Language: English	Non-English publications
Adult population (>18)	Children population (<18) or school-based
Publications that include contexts from low- and middle-income countries	Publications that only include context from high-income countries

For peer review only

Search strategy

The search strategy will cover terms related to the three main components in this study: social networks, NCD prevention community-based programmes, and scale-up/sustainability. Relevant search terms have been developed through review of the literature, author discussion, and in consultation with the team librarian. This helps to better ensure that the review can capture the scope of literature that may use different, but related, terms. Table 3 presents the proposed search terms to be used in the scoping review. The exact search strategy will be adapted to each database.

The following databases will be used for this review: PubMed, Cochrane, Scopus, Web of Science, CINAHL, SocIndex, and IBSS. A complementary search will also be conducted through specialised social network journals: Social Networks, Applied Network Science, and Journal of Social Structure. Grey literature will be identified by reviewing the first three pages of Google and Google Scholar as well as through the IBSS database. Articles that are hand-searched through the references of included publications or suggested through expert opinion will also be included.

Table 3. Proposed search strategy for PubMed

#	Search strategy
	**Community based programmes**
1	(("community-based" OR "community based" OR community OR complex OR collective) AND (intervention OR program* OR trial OR evaluation OR action OR research)) OR ("Community-Based Participatory Research"[Mesh]) OR "Community Participation"[Mesh]
	**Social networks**
2	("social network*" OR "social network analysis" OR "social network intervention*" OR "network analysis" OR "community networks" OR “social relationships” OR “social capital” OR “whole networks”) OR (("Social Network Analysis"[Mesh] OR "Social Networking"[Mesh]) OR ("Community Networks"[Mesh]))
	**Scale-up and sustainability**
3	(("scale-up" OR "scale up" OR sustain* OR implement* OR disseminat*) OR (("Diffusion of Innovation"[Mesh]) OR "Program Evaluation"[Mesh])) OR ("diffusion of innovation" OR "program evaluation")
	**NCD prevention**
4	(Physical activity OR exercise OR diet OR "non communicable" OR chronic OR health promotion OR weight) OR ("Exercise"[Mesh] OR "Diet"[Mesh] OR "Noncommunicable Diseases"[Mesh] OR "Chronic Disease"[Mesh] OR "Health Promotion"[Mesh] OR "Obesity"[Mesh])
	**Setting**
5	((Afghanistan OR Albania OR Algeria OR American Samoa OR Angola OR Armenia OR Azerbaijan OR Bangladesh OR Belarus OR Byelarus OR Belorussia OR Belize OR Benin OR Bhutan OR Bolivia OR Bosnia OR Botswana OR Brazil OR Bulgaria OR Burma OR Burkina Faso OR Burundi OR Cabo Verde OR Cape Verde OR Cambodia OR Cameroon OR Central African Republic OR Chad OR China OR Colombia OR Comoros OR Comores OR Comoro OR Congo OR Costa Rica OR Côte d'Ivoire OR Cuba OR Djibouti OR Dominica OR Dominican Republic OR Ecuador OR Egypt OR El Salvador OR Equatorial Guinea OR Eritrea OR Ethiopia OR Fiji OR Gabon OR Gambia OR Gaza OR Georgia OR Georgia Republic OR Ghana OR Grenada OR

	Grenadines OR Guatemala OR Guinea OR Guinea- Bissau OR Guyana OR Haiti OR Herzegovina OR Hercegovina OR Honduras OR India OR Indonesia OR Iran OR Iraq OR Ivory Coast OR Jamaica OR Jordan OR Kazakhstan OR Kenya OR Kiribati OR Democratic People's Republic of Korea OR Kosovo OR Kyrgyz OR Kirghizia OR Kirghiz OR Kyrgyzstan OR Lao PDR OR Laos OR Lebanon OR Lesotho OR Liberia OR Libya OR Macedonia OR Madagascar OR Malawi OR Malay OR Malaya OR Malaysia OR Maldives OR Mali OR Marshall Islands OR Mauritania OR Mauritius OR Mexico OR Micronesia OR Moldova OR Mongolia OR Montenegro OR Morocco OR Mozambique OR Myanmar OR Namibia OR Nepal OR Nicaragua OR Niger OR Nigeria OR Pakistan OR Palau OR Papua New Guinea OR Paraguay OR Peru OR Philippines OR Principe OR Romania OR Ruanda OR Rwanda OR Samoa OR Sao Tome OR Senegal OR Serbia OR Sierra Leone OR Solomon Islands OR Somalia OR South Africa OR South Sudan OR Sri Lanka OR St Lucia OR St Vincent OR Sudan OR Surinam OR Suriname OR Swaziland OR Syria OR Syrian Arab Republic OR Tajikistan OR Tadzhikistan OR Tajikistan OR Tadzhik OR Tanzania OR Thailand OR Timor OR Togo OR Tonga OR Tunisia OR Turkey OR Turkmen OR Turkmenistan OR Tuvalu OR Uganda OR Ukraine OR Uzbek OR Uzbekistan OR Vanuatu OR Venezuela OR Vietnam OR West Bank OR Yemen OR Zambia OR Zimbabwe) OR (Deprived Countries OR Developing Countries OR Developing Country OR Developing Economies OR Developing Economy OR Developing Nation OR Developing Nations OR Developing World OR LAMI Countries OR LAMI Country OR Less Developed Countries OR Less Developed Country OR Less Developed Economies OR Less Developed Nation OR Less Developed Nations OR Less Developed World OR Lesser Developed Countries OR Lesser Developed Nations OR LMIC OR LMICS OR Low GDP OR Low GNP OR Low Gross Domestic OR Low Gross National OR Low Income OR Lower GDP OR lower gross domestic OR Lower Income OR Middle Income OR Poor Countries OR Poor Country OR Poor Economies OR Poor Economy OR Poor Nation OR Poor Nations OR Poor Population OR Poor Populations OR poor world OR Poorer Countries OR Poorer Economies OR Poorer Economy OR Poorer Nations OR Poorer Population OR Poorer Populations OR Third World OR Transitional Countries OR Transitional Country OR Transitional Economies OR Transitional Economy OR Under Developed Countries OR Under Developed Country OR under developed nations OR Under Developed World OR Under Served Population OR Under Served Populations OR Underdeveloped Countries OR Underdeveloped Country OR underdeveloped economies OR underdeveloped nations OR underdeveloped population OR Underdeveloped World OR Underserved Countries OR Underserved Nations OR Underserved Population OR Underserved Populations)) OR ("Developing Countries"[Mesh]) OR Global South OR Africa OR Latin America OR South America OR Sub-Saharan Africa OR Asia
6	1 AND 2 AND 3 AND 4 AND 5
7	Limit to English language
8	Limit to since year 2000

Study selection

NTA will carry out the searches of the electronic databases. Title and abstracts will be extracted into the reference manager, EndNote, in order to remove all duplicates. NTA will conduct the first stage of screening (titles only) and CF will review the first 10% of titles to test for any reviewer discrepancies. For the second (title and abstracts) and third stage of screening (full text), all publications will be reviewed by two reviewers – NTA and a second reviewer who is independent of the broader research project. This will be done using the Rayyan QCRI systematic review platform, a

For peer review only

web-based tool for systematic review management. Considering the broad scope of expected studies, continuous engagement around discrepancies will be performed to improve reliability and ensure data is not missed.(37, 38) Any discrepancies that cannot be agreed on will be discussed with a third reviewer (CF, EVL, FM, or ZT).

Data extraction and analysis

After full text-review, included articles will be thematically analysed for their network, CBP, and scale-up properties using the Framework Approach.(37) The Framework Approach is a deductive qualitative analysis strategy that seeks to analyse data using pre-set categories. This structured approach is useful in answering specific questions from a diverse body of literature where one can identify themes from the outset while still being flexible and true to the data. There are five stages of data analysis in the framework approach (37):

Familiarisation with the data. The first reviewer (NTA) will familiarise themselves with the data set, noting any recurrent themes.

Identifying a thematic framework. The framework with which to analyse and compare the data set will be based on the MCMO configuration of realist evaluation.(33, 39) Two reviewers (NTA and the independent reviewer) will extract the data using a customised data extraction form (Table 4) to guide the analysis. The data extraction form will be independently piloted by both reviewers on a sub-set of publications, and refined, to improve reliability.(27, 38)

Indexing. Study characteristics to be considered are the author, year of publication, journal/publisher, type of article, the network and actors (resource), the intervention (context), possible mechanisms (reasoning), and the definitions of scale-up and/or sustainability (outcome). These will be extracted onto an Excel spreadsheet. Additional themes that emerge from the data, relevant to the MCMO framework, may also be considered. Studies will be coded and indexed based on these pre-set themes identified. A third reviewer will help to resolve any disagreements.

Charting. The first reviewer (NTA) will collate themes and articles into a final chart in order to easily compare the themes across studies.

Mapping and interpretation. The chart and its themes will be rearranged and analysed based on MCMO configurations. Configurations will be interpreted in line with the research aims.

Table 4. Proposed data extraction form

**Theme**	**Characteristics**
Publication details	Study ID, author, year published, journal/publisher, country, type of article (theoretical vs primary)

For peer review only

Context	Description of CBP, number of components/activities/outcomes, description of community involvement, pertinent contextual factors, CBP theories utilised
Resources	Description of the network structure/intervention, list of actors
Outcomes	Description of the CBP outcomes, description of the scale-up outcomes, vertical or horizontal scale-up, description of sustainability outcomes, theory use, notes on effectiveness
Mechanisms	Possible underlying mechanisms, theory used

Limitations

There are various limitations in this review. Scoping reviews do not provide a critique of the included publications' methodology and so cannot make claims about validity or effectiveness.(27) In addition, the wide range of potential terms used in the literature, such as 'scale-up' vs 'dissemination' vs 'implementation' (15) and the limitation on databases searched may mean that important publications are missed. To mitigate these limitations, there will be continuous engagement with the literature and among authors to refine the terms and multiple reviewers will be used to analyse the publications to increase reliability and credibility of the research.(27, 38) Considering scoping reviews are an iterative process, any revisions to this protocol will be reported to maintain transparency.(38)

Patient and public involvement

No patients or public will be included in formulating or conducting this research as this is a review of already published literature. However, through semi-structured interviews, as part of the broader research project, community and government groups in a middle-income country (South Africa) will receive a chance to comment on the findings and add to and refine the research at a later date.

Ethics and dissemination

Ethical approval will not be sought for this review as no individual-level data or human participants will be involved. The findings from the review will be published in an accredited journal. The PRISMA-ScR checklist will be used to support transparency and guide translation of the review. The findings will also be used to inform the next stages in the broader research project. Findings from this broader research will also be published in a peer-review journal and shared on relevant social media platforms.

References

For peer review only

1. World Health Organization. Tackling NCDs: 'best buys' and other recommended interventions for the prevention and control of noncommunicable diseases. Geneva: World Health Organization; 2017 2017. Contract No.: WHO/NMH/NVI/17.9.
2. Anderson J, Parker W, Steyn N, Grimsrud A, Kolbe-Alexander T, Lambert E, et al. Interventions on diet and physical activity: what works: summary report: World Health Organization; 2009. https://www.who.int/dietphysicalactivity/whatworks/en/
3. Baker PRA, Francis DP, Soares J, Weightman AL, Foster C. Community wide interventions for increasing physical activity. Cochrane Database of Systematic Reviews. 2015(1).
4. Merzel C, D'Afflitti J. Reconsidering community-based health promotion: promise, performance, and potential. American Journal of Public Health. 2003;93(4):557-74.
5. Ansell LH. Community-Based Health Interventions: Past, Present, and Future. In: Steven Whitman AS, and Maureen Benjamins, editor. Urban Health: Combating Disparities with Local Data. Online: Oxford Scholarship Online; 2010.
6. Bruce ML, Smith W, Miranda J, Hoagwood K, Wells KB. Community-based interventions. Mental Health Services Research. 2002;4(4):205-14.
7. McLeroy KR, Norton BL, Kegler MC, Burdine JN, Sumaya CV. Community-based interventions. American Journal of Public Health. 2003;93(4):529-33.
8. Institute of Medicine. Introduction. In: Institute of Medicine, editor. An Integrated Framework for Assessing the Value of Community-Based Prevention. Washington (DC): The National Academies Press; 2012.
9. Jagosh J, Macaulay AC, Pluye P, Salsberg J, Bush PL, Henderson J, et al. Uncovering the benefits of participatory research: Implications of a realist review for health research and practice. Milbank Quarterly. 2012;90(2):311-46.
10. Lepore W, Hall BL, Tandon R. The Knowledge for Change Consortium: a decolonising approach to international collaboration in capacity-building in community-based participatory research. Canadian Journal of Development Studies/Revue canadienne d'études du développement. 2020:1-24.
11. Bauman A, Nutbeam D. Evaluation in a nutshell: a practical guide to the evaluation of health promotion programs: McGraw-Hill; 2013.
12. Ding D, Ramirez Varela A, Bauman AE, Ekelund U, Lee I-M, Heath G, et al. Towards better evidence-informed global action: lessons learnt from the Lancet series and recent developments in physical activity and public health. British Journal of Sports Medicine. 2020;54(8):462-8.
13. Rabkin M, El-Sadr WM. Why reinvent the wheel? Leveraging the lessons of HIV scale-up to confront non-communicable diseases. Global Public Health. 2011;6(3):247-56.
14. Milat AJ, Bauman A, Redman S. Narrative review of models and success factors for scaling up public health interventions. Implementation Science. 2015;10(1):113.
15. McKay H, Naylor P-J, Lau E, Gray SM, Wolfenden L, Milat A, et al. Implementation and scale-up of physical activity and behavioural nutrition interventions: an evaluation roadmap. International Journal of Behavioral Nutrition and Physical Activity. 2019;16(1):102.
16. World Health Organisation. Practical guidance for scaling up health service innovations. Geneva; 2009. https://www.who.int/reproductivehealth/publications/strategic_approach/9789241598521/en/
17. Moore, Mascarenhas A, Bain J, Straus SE. Developing a comprehensive definition of sustainability. Implementation Science. 2017;12(1):110.
18. Luke DA, Stamatakis KA. Systems science methods in public health: dynamics, networks, and agents. Annual Review of Public Health. 2012;33(1):357-76.
19. Robins G. Doing social network research: network-based research design for social scientists. London: Sage; 2015.

20. Borgatti, Mehra A, Brass DJ, Labianca G. Network analysis in the social sciences. Science. 2009;323(5916):892-5.
21. Cross R, Borgatti SP, Parker A. Making invisible work visible: using social network analysis to support strategic collaboration. California Management Review. 2002;44(2):25-46.
22. Hunter RF, Wickramasinghe K, Ergüder T, Bolat A, Arı HO, Yıldırım HH, et al. National action plans to tackle NCDs: role of stakeholder network analysis. BMJ. 2019;365:l1871.
23. Valente TW. Network interventions. Science. 2012;337(6090):49-53.
24. Valente TW, Palinkas LA, Czaja S, Chu K-H, Brown CH. Social network analysis for program implementation. PloS One. 2015;10(6):e0131712-e.
25. Hunter RF, De la Haye K, Murray J, Badham J, Valente T, Clarke M, et al. Social network interventions for health behaviours and outcomes: a systematic review and meta-analysis. PLoS Med. 2019;16(9):e1002890.
26. Munn Z, Peters MDJ, Stern C, Tufanaru C, McArthur A, Aromataris E. Systematic review or scoping review? Guidance for authors when choosing between a systematic or scoping review approach. BMC Medical Research Methodology. 2018;18(1):143.
27. Levac D, Colquhoun H, O'Brien KK. Scoping studies: advancing the methodology. Implementation Science. 2010;5(1):69.
28. Arksey H, O'Malley L. Scoping studies: towards a methodological framework. International Journal of Social Research Methodology. 2005;8(1):19-32.
29. Tricco AC, Lillie E, Zarin W, O'Brien KK, Colquhoun H, Levac D, et al. PRISMA extension for scoping reviews (PRISMA-ScR): checklist and explanation. Annals of Internal Medicine. 2018;169(7):467-73.
30. Fynn JF, Hardeman W, Milton K, Jones AP. A scoping review of evaluation frameworks and their applicability to real-world physical activity and dietary change programme evaluation. BMC Public Health. 2020;20(1):1000.
31. Moore, Audrey S, Barker M, Bond L, Bonell C, Hardeman W, et al. Process evaluation of complex interventions: Medical Research Council guidance. BMJ. 2015;350(mar19 6):h1258.
32. Westhorp G. Realist impact evaluation: an introduction. London: Overseas Development Institute. 2014:1-12.
33. Dalkin SM, Greenhalgh J, Jones D, Cunningham B, Lhussier M. What's in a mechanism? Development of a key concept in realist evaluation. Implementation Science. 2015;10(1):49.
34. World Health Organisation. Noncommunicable diseases. 2021. https://www.who.int/news-room/fact-sheets/detail/noncommunicable-diseases [Accessed 28 April 2021].
35. Mozaffarian D, Afshin A, Benowitz NL, Bittner V, Daniels SR, Franch HA, et al. Population approaches to improve diet, physical activity, and smoking habits. Circulation. 2012;126(12):1514-63.
36. World Bank. World Bank Country and Lending Groups 2021. https://datahelpdesk.worldbank.org/knowledgebase/articles/906519-world-bank-country-and-lending-groups [Accessed 28 April 2021].
37. Pope C, Ziebland S, Mays N. Qualitative research in health care. analysing qualitative data. BMJ (Clinical Research Edition). 2000;320(7227):114-6.
38. Gilson L. Health policy and system research: a methodology reader: the abridged version. World Health Organization; 2013. https://www.who.int/alliance-hpsr/resources/reader/en/
39. Mukumbang FC, Marchal B, Van Belle S, van Wyk B. A realist approach to eliciting the initial programme theory of the antiretroviral treatment adherence club intervention in the Western Cape Province, South Africa. BMC Medical Research Methodology. 2018;18(1):47.

Footnotes

For peer review only

Contributors All authors conceptualised the review. NTA led the writing of the protocol. CF, EVL, FM, and ZT provided feedback and improvements. All authors read and approved the final manuscript. Acknowledgement of contribution for this protocol should also be given the University of Cape Town librarian team.

Funding NTA is supported through a University of Bristol QR GCRF grant. Bristol's QR GCRF strategy funding is awarded to the University by Research England. Additional funding was provided by philanthropic donations from the University of Bristol's Alumni and Friends. The funders do not have any involvement in data collection, data analysis, or data interpretation.

Competing interests None declared.

Patient consent Not required.

Provenance and peer review Not commissioned; externally peer reviewed.

Copyright The Corresponding Author has the right to grant on behalf of all authors and does grant on behalf of all authors, a world-wide licence to the Publishers and its licencees in perpetuity, in all forms, formats and media (whether known now or created in the future), to i) publish, reproduce, distribute, display and store the Contribution, ii) translate the Contribution into other languages, create adaptations, reprints, include within collections and create summaries, extracts and/or, abstracts of the Contribution, iii) create any other derivative work(s) based on the Contribution, iv) to exploit all subsidiary rights in the Contribution, v) the inclusion of electronic links from the Contribution to third party material where—ever it may be located; and, vi) licence any third party to do any or all of the above.

For peer review only

BMJ Open

Using social networks to scale-up and sustain community-based programmes to improve physical activity and diet in low- and middle-income countries: a scoping review protocol.

Journal:	*BMJ Open*
Manuscript ID	bmjopen-2021-053586.R1
Article Type:	Protocol
Date Submitted by the Author:	24-Jul-2021
Complete List of Authors:	Abrahams, Nina; University of Bristol, Exercise, Nutrition, and Health Sciences; Faculty of Health Sciences University of Cape Town, Health through Physical Activity Lifestyle and Sport Research Centre<br>Lambert, Estelle; University of Cape Town, Health through Physical Activity Lifestyle and Sport Research Centre<br>Marais, Frederick; Western Cape Government, Department of Health ; North-West University, Africa Unit for Transdisciplinary Health Research<br>Toumpakari, Zoi; University of Bristol, Centre for Exercise, Nutrition, and Health Sciences<br>Foster, Charlie ; University of Bristol, Centre for Exercise, Nutrition, and Health Sciences
**Primary Subject Heading**:	Public health
Secondary Subject Heading:	Qualitative research, Research methods
Keywords:	PUBLIC HEALTH, Quality in health care < HEALTH SERVICES ADMINISTRATION & MANAGEMENT, QUALITATIVE RESEARCH

SCHOLARONE™
Manuscripts

I, the Submitting Author has the right to grant and does grant on behalf of all authors of the Work (as defined in the below author licence), an exclusive licence and/or a non-exclusive licence for contributions from authors who are: i) UK Crown employees; ii) where BMJ has agreed a CC-BY licence shall apply, and/or iii) in accordance with the terms applicable for US Federal Government officers or employees acting as part of their official duties; on a worldwide, perpetual, irrevocable, royalty-free basis to BMJ Publishing Group Ltd ("BMJ") its licensees and where the relevant Journal is co-owned by BMJ to the co-owners of the Journal, to publish the Work in this journal and any other BMJ products and to exploit all rights, as set out in our licence.

The Submitting Author accepts and understands that any supply made under these terms is made by BMJ to the Submitting Author unless you are acting as an employee on behalf of your employer or a postgraduate student of an affiliated institution which is paying any applicable article publishing charge ("APC") for Open Access articles. Where the Submitting Author wishes to make the Work available on an Open Access basis (and intends to pay the relevant APC), the terms of reuse of such Open Access shall be governed by a Creative Commons licence – details of these licences and which Creative Commons licence will apply to this Work are set out in our licence referred to above.

Other than as permitted in any relevant BMJ Author's Self Archiving Policies, I confirm this Work has not been accepted for publication elsewhere, is not being considered for publication elsewhere and does not duplicate material already published. I confirm all authors consent to publication of this Work and authorise the granting of this licence.

For peer review only

Using social networks to scale-up and sustain community-based programmes to improve physical activity and diet in low- and middle-income countries: a scoping review protocol.

Nina Abrahams*[1,2], Estelle V. Lambert[2], Frederick Marais[3,4], Zoi Toumpakari[1], Charlie Foster[1]

[1] Centre for Exercise, Nutrition and Health Sciences, School for Policy Studies, Faculty of Social Sciences and Law, University of Bristol, Bristol, United Kingdom
[2] Health through Physical Activity Lifestyle and Sport Research Centre, Lifestyle and Sport, Division of Exercise Science and Sports Medicine, Department of Human Biology, Faculty of Health Sciences, University of Cape Town, Cape Town, South Africa
[3] Increasing Wellness, Department of Health, Western Cape Government, South Africa
[4] Africa Unit for Transdisciplinary Health Research, North-West University, South Africa

*Correspondence to Nina Abrahams; ntabrahams@gmail.com

For peer review only

Abstract

Introduction The World Health Organisation endorses community-based programmes as a cost-effective, feasible, and a 'best buy' in the prevention and management of non-communicable diseases. These programmes are particularly successful when the community actively participates in its design, implementation, and evaluation. However, they may be only useful insofar as they can be scaled-up and sustained in some meaningful way. Social network research may serve as an important tool for determining the underlying mechanisms that contribute to this process. The aim of this planned scoping review is to map and collate literature on the role of social networks in scaling-up and sustaining community-based physical activity and diet programmes in low- and middle-income countries

Methods and analysis This scoping review protocol has been planned around the Arksey and O'Malley framework and its enhancement. Inclusion criteria are peer-reviewed articles and grey literature exploring the role of social networks in the scale-up and/or sustainability of NCD prevention community-based programmes in adult populations. Studies must have been published since 2000 and based in a low- or middle-income country. The following databases will be used for this review: PubMed, Cochrane, Scopus, Web of Science, CINAHL, SocIndex, the International Bibliography of the Social Sciences, Google, and Google Scholar. Books, conference abstracts, and research focused only on children will be excluded. Two reviewers will independently select and extract eligible studies. Included publications will be thematically analysed using the Framework Approach.

Ethics and dissemination Ethical approval will not be sought for this review as no individual-level data or human participants will be involved. This protocol is registered on the Open Science Framework (https://doi.org/10.17605/OSF.IO/KG7TX). The findings from the review will be published in an accredited journal. The PRISMA-ScR checklist will be used to support transparency and guide translation of the review.

Strengths and limitations of this study
➢ This study will help fill a research gap of how social networks are used to scale-up and sustain physical activity and diet community-based programmes in low- and middle-income countries.
➢ This protocol is based on a widely-used framework for scoping reviews and will make use of iterative steps to continously improve rigour.
➢ Representatives of the public will have a chance to comment on and refine the study findings.
➢ Although comprehensive, the protocol has limitations with regards to search terms, language, and databases used.
➢ Quality of evidence will not be evaluated in this scoping review.

For peer review only

Introduction

The World Health Organisation endorses a set of 16 programmes that are shown to be cost-effective, feasible, and so 'best buys' in the prevention and management of non-communicable diseases [NCDs] at an individual and population level.(1) One of the 'best buy' programmes for both physical activity and diet are community-based awareness, educational, and behaviour change programmes. These community-based programmes [CBPs] vary in practice and so do not have a set definition but refer broadly to programmes that target and engage a defined population in activities ranging widely from group-based interventions or mass media campaigns to environmental, structural or policy changes that are adapted to, set in, and ideally delivered by the community for that community.(2-9) Programmes wherein communities actively participate and lead in design, implementation, and evaluation have been shown to be particularly creative, sustainable, and effective in improving health outcomes and developing longer-term confidence and capacity building within communities.(7, 9-12) These programmes are not clinical or pharmaceutical-based but are more focused on awareness, education, and creating a supportive environment for behaviour change, social change, and community development.(1, 2, 11)

One could argue that CBPs are only truly useful insofar as they can be scaled-up and sustained in some meaningful way. If the CBP does not reach enough people, then their effects are spread thin and the CBP has less chance of making a sustainable and significant impact.(2, 3, 5, 13-15) Scale-up of an intervention has been defined as *"deliberate efforts to increase the impact of successfully tested health innovations so as to benefit more people and to foster policy and programme development on a lasting basis." (16)* This can refer to the intervention or programme being introduced to a greater number of its target population, being adapted for other populations, or becoming increasingly ratified at a

national or international policy level.(14, 16-19) Sustainability, a core component of scaling-up, has been defined as when *“the program continues to be delivered and/or individual behaviour change is maintained; the program and individual behaviour change may evolve or adapt while continuing to produce benefits for individuals/systems, after a defined period of time.”*(20) This refers to the political and institutional adoption of the programme beyond the initial funding or starting team.(16)

There is, however, limited research on how to effectively scale-up and sustain complex community-based NCD prevention programmes.(17, 18) This dearth of literature is particularly pertinent in low- and middle-income countries [LMICs]. For example, in 2016, Reis et al. (21) conducted a systematic review on scaling-up physical activity interventions. The authors only identified 16 scaled-up interventions in peer-reviewed literature, of which only two were based in an LMIC. While some evidence of scale-up does exist in LMICs, they tend to significantly focus on HIV/AIDs, maternal health, and infectious diseases compared to NCD prevention.(22, 23)

One potentially useful tool for improving the theory and practice of the scale-up and sustainability of complex CBPs is social network research [SNR]. SNR is research that examines the relationship between actors (individuals or organisations) in a system.(24, 25) The aim of SNR is to identify who is in a particular network, what their attributes are, and who they are connected to in the network and then to visually plot these relationship on a network graph.(25) There are various ways in which network structures can then be analysed.(26-28) For example, one can calculate the density of the network (how many ties/relationships there are between actors) where a high degree of ties to one stakeholder may indicate that they are considered a leader. Alternatively, there may be several densely tied sub-groups within a bigger network with bridging actors who connect these clusters. Once one understands how the network is functioning, one can then try and utilise these connections by generating strategies for greater and more efficient network reach and sustainability.(25, 29, 30) Valente (29) suggests four main ‘network interventions’: identifying key actors, identifying and shifting the actions of sub-clusters at a time, stimulating peer-to-peer influence, and altering the network (removing or adding actors into key network positions). In 2019, Hunter et al. (31) conducted a systematic review and meta-analysis of randomised controlled trials to identify the effectiveness of social network interventions over a range of health behaviours and outcomes. The pooled evidence indicates that social network interventions result in improved health outcomes and are particularly useful at reaching and retaining underserved populations. However, of the 37 included interventions only six studies were based in a LMIC, none of which took place in Africa.

Broader research

For peer review only - http://bmjopen.bmj.com/site/about/guidelines.xhtml

To fill this gap, the authors (NTA, CF, EVL, FM, ZT) are conducting research to understand and create theory of the various attributes of social networks that may impact or influence the successful implementation, scale-up, and sustainability of community-based, NCD prevention, programmes. The research consists of this scoping review, a social network analysis of a case study CBP, and realist interviews with stakeholders of the CPB.

The CPB case study is a government-run healthy lifestyles partnership initiative in South Africa, the WesternCape on Wellness [WoW!].(9) The programme started in 2015 and aims to prevent, reduce, and better manage NCDs by using peer networks and partnerships to promote healthy eating, healthy weight, as well as increase health-related physical activity, social connectedness, and mental wellness. Currently, WoW! has most focused on physical activity and diet in adult populations and so for relevancy and scope manageability this will also be the focus of the planned scoping review.

Scoping review research aim and questions

The aim of this scoping review is to map and collate literature on the role of social networks for scaling-up and sustaining NCD prevention physical activity and diet CBPs in LMICs. The findings of this review will be used to determine the current scope of research, help to identify gaps in the literature, and support the development of an initial programme theory as part of the broader research project aims.

The overarching research question is: *Is there research on social networks within scale-up studies of community-based physical activity and diet programmes in low- and middle-income countries? And if so, what is the nature of the role of social networks?*

The sub-questions that will guide the scoping review are presented in table 1.

Table 1. Scoping review questions

**Descriptive**	What is the volume of publications?
	What are the research designs of the publications?
	What is the geographical scope of the publications?
	Who are the publication authors?
**Social networks**	What types of networks and/or network interventions are described in the publications? What are they used for?
	Who is involved in the network(s)?
	What value, if any, do social networks bring to community-based programmes?
**Community-based programmes**	What types of community-based programmes are covered?
	What activities are included in the community-based programmes? Who is included? (age, sex, gender, health and economic status).

For peer review only

	Who is implementing these programmes? What settings are used for the programmes?
	What theories/theoretical approaches underpin the community-based programmes?
**Scale-up and sustainability**	What scale-up/sustainability theories are utilised in the publications?
	How is scale-up and/or sustainability conceptualised or operationalised?
**Mechanisms**	Are any potential mechanisms of scale-up and sustainability explored in the publications?

Methods and analysis

Scoping reviews are useful for determining the broad scale and range of a body of literature and exploring key factors related to a particular concept of interest.(32, 33) This is particularly useful when there is a paucity of evidence in that field.(33) Scoping reviews are not intended for answering a specific question in detail but rather to gain a broad understanding of a particular topic.

This protocol has been planned around the methodological framework for scoping reviews outlined by the Arksey and O'Malley framework (34) as well as its enhancement.(33) The method and final reporting will also be based on the checklist provided by the Preferred Reporting Items for Systematic Reviews and Meta-analyses extension for Scoping Reviews [PRISMA-ScR].(35) These frameworks acknowledge that scoping reviews are an iterative process and that continuous engagement and refinement is needed to retrieve relevant publications and analyse reliably.(33) Any iterations or deviations from this protocol will be reported in the final findings' publication.

Conceptual framework

This planned scoping review will form part of a larger research project that is framed by a realist evaluation. Realist evaluation is a theory-based evaluation strategy that aims to answer the question 'what works, how or why does this work, for whom, and in what circumstances?'.(36-39) Namely, a theory about how a programme is thought to operate is generated based on known literature, this initial theory is then tested on a real-world case study and, based on the results, the programme theory is refined. The programme theory is built using the structure: resource mechanisms-context-reasoning mechanisms-outcomes. Otherwise denoted as 'M(Resources)+C→M(Reasoning)=O' or [MCMO].(39)

In this research, the resource mechanism will be the social network structure of a public health intervention, such as, who the actors are, how many sub-groups there are in the network, and whether any network interventions have been used to alter the network. The context is the

For peer review only

intervention itself and the social and geographical environment within which it operates. The outcome will be increased scale-up and sustainability. Variables are defined as:

1. Community based programmes
   - CBPs include any NCD prevention programmes or interventions that aim to change a defined population's psychological thinking, social interactions, or behaviours. They exclude interventions on a clinical or pharmaceutical level.
     - A broad definition of NCD prevention will be used to include any programme that promotes health and prevents non-communicable diseases.(40) Activities should include promotion of physical activity, healthy diets, or reducing sendentary behaviour.
     - CBPs may include programmes that use a defined community as a setting or programmes that include the community in the design, running, and evaluation of the intervention.(7)
     - Activities and outcomes of CBPs may include, but are not limited to, mass media education and awareness campaigns, group-based education or activity classes, individual motivational interviewing or brief behaviour change counselling, changing built environments, improving access to resources, community capacity building and empowerment, and improved social cohesion.(2, 10, 11, 41)
2. Scale-up and/or sustainability
   - Scale-up, namely a deliberate intention of growth, must be emphasised by the authors as an outcome or variable of interest. It can be horizontal (different populations or more of the population, or increasing programme innovation) or vertical (embedded in policy) scale-up.(18)
   - AND/OR
   - Sustainability must be emphasised by the authors as an outcome or variable of interest. It can include any interest in the programme and its effects continuing after initial implementation with specific interest in institutional and policy ratification.(20)
3. Social networks
   - A broad definition of social networks will be used in line with previous systematic reviews of social network interventions.(31) Social networks encompass any social interaction or relationship between people or organisations, in vivo or online.
   - To be included a publication must have measured or considered these relationships in some aspect of the intervention design or delivery. This includes recognising an existing

For peer review only

social network as being important to the success of the intervention (social network) or actively manipulating a network as part of the scale-up or implementation strategy (social network intervention).

4. Actors
   - ➢ Any of the people or organisations mentioned that play a role in the CBP (such as intervention developers, implementers, community members, participants, funders, policy makers).
5. Reasoning mechanisms
   - ➢ Underlying social or psychological responses that potentially foster or inhibit the outcome of scale-up and/or sustainability (such as trust or communication patterns).
6. Low- and middle-income countries
   - ➢ Countries that are low-income, lower-middle income, or upper-middle income economies as categorised by the World Bank in 2021.(42)

Inclusion and exclusion criteria

To be included, articles must explore network, context, and outcome components. Namely they must explore the role that the structure of a social network may have to influence scaling-up and/or sustaining community-based programmes. At least two of these components should be highlighted in the abstract or summary of the publications to be considered for further review. Included articles can be peer-reviewed frameworks, theoretical papers, reviews, or primary (quantitative, qualitative, or mixed method) studies or grey literature, published in English since 2000. This start date is based on the rapid rise of CBP and scale-up literature since 2000 on the International Bibliography of the Social Sciences [IBSS] database. The cut-off date will be reflected in the final scoping review report. Inclusion population are human adults (19-64) and older adults (>65). If the publication has a wide age range, then the mean age should be >18 or more than 50% of the participants or beneficiaries should fall within the age range. Articles will be included if they have a wide age range but have analysed adult and child populations separately. Child or school-based only programmes are not included in the review at this stage as the broader research project focuses on adult population networks. In addition, only publications that include low- and middle-income contexts or considerations in their analysis will be considered. Excluded studies include conference abstracts, protocols, books, any outputs not published in English, and publications only focused on high-income countries (Table 2).

Considering the expected dearth of literature, the planned scoping review will be kept broad. However, as this review follows an iterative process, inclusion and exclusion criteria may be further

For peer review only

refined after the initial retrieval and review of abstracts to closer reflect the larger research project aims.(33) For example, CBPs may be further refined to only include multi-component (complex) programmes. Namely, programmes with more than one activity or component.(3, 37) In addition, articles may be refined to only include CBPs wherein the community has been intimately involved in developing and implementing the intervention.(7, 10) Any adaptions to the inclusion criteria will be reported in the final publication.

Table 2. Inclusion and exclusion criteria

**Inclusion criteria**	**Exclusion criteria**
Articles exploring the role of social networks in the scale-up and/or sustainability of NCD prevention community-based programmes	Books, book chapters, book reviews
Peer-reviewed articles, including original research, reviews, commentaries, and opinion pieces	Conference proceedings, dissertations/theses, and abstracts
Grey literature (e.g., institution reports, government documents)	Protocols
Indexed in PubMed, CINAHL, Scopus, Web of Science, Cochrane, IBSS, Google (Scholar)	Website, newspaper, and social media content
Published from year 2000 onwards	Published before 01 Jan 2000
Language: English	Non-English publications
Adult population (>18)	Children population (<18) or school-based
Publications that include contexts from low- and middle-income countries	Publications that only include context from high-income countries

Search strategy

The search strategy will cover terms related to the three main components in this study: social networks, NCD prevention community-based programmes, and scale-up/sustainability. Relevant search terms have been developed through review of the literature, author discussion, and in consultation with the team librarian. This helps to better ensure that the review can capture the scope of literature that may use different, but related, terms. Supplementary additional file 1 presents the search terms to be used in the scoping review.

The following databases will be used for this review: PubMed, Cochrane, Scopus, Web of Science, CINAHL, SocIndex, and IBSS. A complementary search will also be conducted through specialised social network journals: Social Networks, Applied Network Science, and Journal of Social Structure. Grey literature will be identified by reviewing the first three pages of Google and Google Scholar as well as through the IBSS database. Articles that are hand-searched through the references of included publications or suggested through expert opinion will also be included.

For peer review only - http://bmjopen.bmj.com/site/about/guidelines.xhtml

Study selection

NTA will carry out the searches of the electronic databases. Title and abstracts will be extracted into the reference manager, EndNote, in order to remove all duplicates. NTA will conduct the first stage of screening (titles only) and CF will review the first 10% of titles to test for any reviewer discrepancies. For the second (title and abstracts) and third stage of screening (full text), 50% of the publications will be reviewed by two reviewers – NTA and a second reviewer who is independent of the broader research project. This will be done using the Rayyan QCRI systematic review platform, a web-based tool for systematic review management. If there are no major conflicts then the remaining publications will be reviewed by NTA only; however, if there are major conflicts then all publications will be independently reviewed by two reviewers. Considering the broad scope of expected studies, continuous engagement around discrepancies will be performed to improve reliability and ensure data is not missed.(43, 44) Any discrepancies that cannot be agreed on will be discussed with a third reviewer (CF, EVL, FM, or ZT).

Data extraction and analysis

After full text-review, included articles will be thematically analysed for their network, CBP, and scale-up properties using the Framework Approach.(43) The Framework Approach is a deductive qualitative analysis strategy that seeks to analyse data using pre-set categories. This structured approach is useful in answering specific questions from a diverse body of literature where one can identify themes from the outset while still being flexible and true to the data. There are five stages in the framework approach (43):

Data extraction

1) Familiarisation with the data: The first reviewer (NTA) will familiarise themselves with the data set, noting any recurrent themes and gaining an overall picture of included publication.

2) Identifying a thematic framework: The framework with which to analyse and compare the data set will be based on the MCMO configuration of realist evaluation.(39, 45) Two reviewers (NTA and the independent reviewer) will extract the data using a customised data extraction form (Table 3) to guide the analysis. The data extraction form is based on the scoping review questions. It will be independently piloted by both reviewers on a sub-set of publications, and refined, to improve reliability.(33, 44)

3) Indexing: Study characteristics to be considered are the author, year of publication, journal/publisher, type of article, the network and actors (resource), the intervention (context), possible mechanisms (reasoning), and the definitions of scale-up and/or sustainability (outcome).

For peer review only

These will be extracted onto an Excel spreadsheet. Additional themes that emerge from the data, relevant to the MCMO framework, may also be considered. Studies will be coded and indexed based on these pre-set themes identified. A third reviewer will help to resolve any disagreements.

Table 3. Planned data extraction form

**Theme**	**Characteristics**
Publication details	Study ID, author, year published, journal/publisher, country, type of article (theoretical vs primary)
Context	Description of CBP, number of components/activities/outcomes, description of community involvement, pertinent contextual factors, CBP theories utilised
Resources	Description of the network structure/intervention, list of actors
Outcomes	Description of the CBP outcomes, description of the scale-up outcomes, vertical or horizontal scale-up, description of sustainability outcomes, theory use, notes on effectiveness
Mechanisms	Possible underlying mechanisms, theory used

Data analysis

4) Charting: The first reviewer (NTA) will collate themes and articles into a final chart in order to easily compare the themes across studies.

5) Mapping and interpretation: The chart and its themes will be rearranged and thematically analysed based on MCMO configurations. Multiple theories based on possible network, programme, underlying mechanisms, and scale-up/sustainability configurations will be collated to develop an initial programme theory.(38) This theory will be tested and refined as part of the broader research aims.

Limitations

There are various limitations in this review. Scoping reviews do not provide a critique of the included publications' methodology and so cannot make claims about validity or effectiveness.(33) Publications in a language other than English are not included as this is beyond the timeline and scope of this review. In addition, the wide range of potential terms used in the literature, such as 'scale-up' vs 'dissemination' vs 'implementation'(18) 'scalability' and 'spread'(12) and the limitation on databases searched may mean that publications are missed. To mitigate these limitations, there will be continuous engagement with the literature and among authors to refine the terms and multiple reviewers will be used to analyse the publications to increase reliability and credibility of the research.(33, 44) Considering scoping reviews are an iterative process, any revisions to this

For peer review only

protocol will be reported to maintain transparency.(44) Limitations and suggestions for further research will be indicated in the dissemination of findings.

Patient and public involvement

No patients or public will be included in formulating or conducting this research as this is a review of already published literature. However, through semi-structured interviews, as part of the broader research project, community and government groups in a middle-income country (South Africa) will receive a chance to comment on the findings and add to and refine the research at a later date.

Ethics and dissemination

Ethical approval will not be sought for this review as no individual-level data or human participants will be involved. This protocol was registered in July 2021 on the Open Science Framework (https://doi.org/10.17605/OSF.IO/KG7TX). The findings from the review will be published in an accredited journal. The PRISMA-ScR checklist will be used to support transparency and guide translation of the review. The findings will also be used to inform the next stages in the broader research project. Findings from this broader research will also be published in a peer-review journal and shared on relevant social media platforms.

References

1. World Health Organization. Tackling NCDs: 'best buys' and other recommended interventions for the prevention and control of noncommunicable diseases. Geneva: World Health Organization; 2017. Contract No.: WHO/NMH/NVI/17.9.
2. Anderson J, Parker W, Steyn N, Grimsrud A, Kolbe-Alexander T, Lambert E, et al. Interventions on diet and physical activity: what works: summary report. Geneva: World Health Organization; 2009. Available from https://www.who.int/dietphysicalactivity/whatworks/en/
3. Baker PRA, Francis DP, Soares J, Weightman AL, Foster C. Community wide interventions for increasing physical activity. Cochrane Database of Systematic Reviews. 2015(1).
4. Merzel C, D'Afflitti J. Reconsidering community-based health promotion: promise, performance, and potential. American Journal of Public Health. 2003;93(4):557-74.
5. Ansell LH. Community-based health interventions: past, present, and future. In: Steven Whitman AS, and Maureen Benjamins, editor. Urban health: combating disparities with local data. Online: Oxford Scholarship Online; 2010.
6. Bruce ML, Smith W, Miranda J, Hoagwood K, Wells KB. Community-based interventions. Mental Health Services Research. 2002;4(4):205-14.
7. McLeroy KR, Norton BL, Kegler MC, Burdine JN, Sumaya CV. Community-based interventions. American Journal of Public Health. 2003;93(4):529-33..
8. Institute of Medicine. Introduction. In: Institute of Medicine, editor. An integrated framework for assessing the value of community-based prevention. Washington (DC): The National Academies Press; 2012.
9. Wellness Team. Annual community report. Western Cape: Western Cape Government; 2020.

For peer review only

10. Jagosh J, Macaulay AC, Pluye P, Salsberg J, Bush PL, Henderson J, et al. Uncovering the benefits of participatory research: Implications of a realist review for health research and practice. Milbank Quarterly. 2012;90(2):311-46.
11. Lepore W, Hall BL, Tandon R. The knowledge for change consortium: a decolonising approach to international collaboration in capacity-building in community-based participatory research. Canadian Journal of Development Studies/Revue canadienne d'études du développement. 2020:1-24.
12. Ben Charif A, Plourde KV, Guay-Bélanger S, Zomahoun HTV, Gogovor A, Straus S, et al. Strategies for involving patients and the public in scaling-up initiatives in health and social services: protocol for a scoping review and Delphi survey. Systematic Reviews. 2021;10(1):55.
13. Bauman A, Nutbeam D. Evaluation in a nutshell: a practical guide to the evaluation of health promotion programs. McGraw-Hill; 2013.
14. Ding D, Ramirez Varela A, Bauman AE, Ekelund U, Lee I-M, Heath G, et al. Towards better evidence-informed global action: lessons learnt from the Lancet series and recent developments in physical activity and public health. British Journal of Sports Medicine. 2020;54(8):462-8.
15. Rabkin M, El-Sadr WM. Why reinvent the wheel? Leveraging the lessons of HIV scale-up to confront non-communicable diseases. Global Public Health. 2011;6(3):247-56.
16. World Health Organization. Nine steps for developing a scaling-up strategy. Geneva: World Health Organization; 2010.
17. Milat AJ, Bauman A, Redman S. Narrative review of models and success factors for scaling up public health interventions. Implementation Science. 2015;10(1):113.
18. McKay H, Naylor P-J, Lau E, Gray SM, Wolfenden L, Milat A, et al. Implementation and scale-up of physical activity and behavioural nutrition interventions: an evaluation roadmap. International Journal of Behavioral Nutrition and Physical Activity. 2019;16(1):102.
19. World Health Organisation. Practical guidance for scaling up health service innovations. Geneva: World Health Organisation; 2009.
20. Moore, Mascarenhas A, Bain J, Straus SE. Developing a comprehensive definition of sustainability. Implementation Science. 2017;12(1):110.
21. Reis RS, Salvo D, Ogilvie D, Lambert EV, Goenka S, Brownson RC, et al. Scaling up physical activity interventions worldwide: stepping up to larger and smarter approaches to get people moving. The Lancet. 2016;388(10051):1337-48.
22. Bulthuis SE, Kok MC, Raven J, Dieleman MA. Factors influencing the scale-up of public health interventions in low-and middle-income countries: a qualitative systematic literature review. Health Policy and Planning. 2020;35(2):219-34.
23. Mangham LJ, Hanson K. Scaling up in international health: what are the key issues? Health Policy and Planning. 2010;25(2):85-96.
24. Luke DA, Stamatakis KA. Systems science methods in public health: dynamics, networks, and agents. Annual Review of Public Health. 2012;33(1):357-76.
25. Robins G. Doing social network research: network-based research design for social scientists. London: Sage; 2015.
26. Borgatti, Mehra A, Brass DJ, Labianca G. Network analysis in the social sciences. Science. 2009;323(5916):892-5.
27. Cross R, Borgatti SP, Parker A. Making invisible work visible: using social network analysis to support strategic collaboration. California Management Review. 2002;44(2):25-46.
28. Hunter RF, Wickramasinghe K, Ergüder T, Bolat A, Arı HO, Yıldırım HH, et al. National action plans to tackle NCDs: role of stakeholder network analysis. BMJ. 2019;365:l1871..
29. Valente TW. Network interventions. Science. 2012;337(6090):49-53.
30. Valente TW, Palinkas LA, Czaja S, Chu K-H, Brown CH. Social network analysis for program implementation. PloS One. 2015;10(6):e0131712-e.

For peer review only

31. Hunter RF, De la Haye K, Murray J, Badham J, Valente T, Clarke M, et al. Social network interventions for health behaviours and outcomes: a systematic review and meta-analysis. PLoS Med. 2019;16(9):e1002890.
32. Munn Z, Peters MDJ, Stern C, Tufanaru C, McArthur A, Aromataris E. Systematic review or scoping review? Guidance for authors when choosing between a systematic or scoping review approach. BMC Medical Research Methodology. 2018;18(1):143.
33. Levac D, Colquhoun H, O'Brien KK. Scoping studies: advancing the methodology. Implementation Science. 2010;5(1):69.
34. Arksey H, O'Malley L. Scoping studies: towards a methodological framework. International Journal of Social Research Methodology. 2005;8(1):19-32.
35. Tricco AC, Lillie E, Zarin W, O'Brien KK, Colquhoun H, Levac D, et al. PRISMA extension for scoping reviews (PRISMA-ScR): checklist and explanation. Annals of Internal Medicine. 2018;169(7):467-73.
36. Fynn JF, Hardeman W, Milton K, Jones AP. A scoping review of evaluation frameworks and their applicability to real-world physical activity and dietary change programme evaluation. BMC Public Health. 2020;20(1):1000.
37. Moore, Audrey S, Barker M, Bond L, Bonell C, Hardeman W, et al. Process evaluation of complex interventions: Medical Research Council guidance. BMJ. 2015;350:h1258.
38. Westhorp G. Realist impact evaluation: an introduction. London: Overseas Development Institute. 2014:1-12.
39. Dalkin SM, Greenhalgh J, Jones D, Cunningham B, Lhussier M. What's in a mechanism? Development of a key concept in realist evaluation. Implementation Science. 2015;10(1):49.
40. World Health Organisation. Noncommunicable diseases [Internet]. World Health Organisation; 2021 [cited 2021 April 28]. Available from https://www.who.int/news-room/fact-sheets/detail/noncommunicable-diseases
41. Mozaffarian D, Afshin A, Benowitz NL, Bittner V, Daniels SR, Franch HA, et al. Population approaches to improve diet, physical activity, and smoking habits. Circulation. 2012;126(12):1514-63.
42. World Bank. World Bank Country and Lending Groups [Internet]. World Bank; 2021 [cited 2021 April 28]. Available from https://datahelpdesk.worldbank.org/knowledgebase/articles/906519-world-bank-country-and-lending-groups
43. Pope C, Ziebland S, Mays N. Qualitative research in health care. analysing qualitative data. BMJ (Clinical Research Edition). 2000;320(7227):114-6.
44. Gilson L. Health policy and system research: a methodology reader: the abridged version. Geneva: World Health Organization; 2013. Available from https://www.who.int/alliance-hpsr/resources/reader/en/
45. Mukumbang FC, Marchal B, Van Belle S, van Wyk B. A realist approach to eliciting the initial programme theory of the antiretroviral treatment adherence club intervention in the Western Cape Province, South Africa. BMC Medical Research Methodology. 2018;18(1):47.

For peer review only

Footnotes

Contributors All authors conceptualised the review. NTA led the writing of the protocol. CF, EVL, FM, and ZT provided feedback and improvements. All authors read and approved the final manuscript. Acknowledgement of contribution for this protocol should also be given the University of Cape Town librarian team.

Funding NTA is supported through a University of Bristol QR GCRF grant. Bristol's QR GCRF strategy funding is awarded to the University by Research England. Additional funding was provided by

philanthropic donations from the University of Bristol's Alumni and Friends. The funders do not have any involvement in data collection, data analysis, or data interpretation.

Competing interests None declared.

Patient consent Not required.

Provenance and peer review Not commissioned; externally peer reviewed.

Copyright The Corresponding Author has the right to grant on behalf of all authors and does grant on behalf of all authors, a world-wide licence to the Publishers and its licencees in perpetuity, in all forms, formats and media (whether known now or created in the future), to i) publish, reproduce, distribute, display and store the Contribution, ii) translate the Contribution into other languages, create adaptations, reprints, include within collections and create summaries, extracts and/or, abstracts of the Contribution, iii) create any other derivative work(s) based on the Contribution, iv) to exploit all subsidiary rights in the Contribution, v) the inclusion of electronic links from the Contribution to third party material where—ever it may be located; and, vi) licence any third party to do any or all of the above.

For peer review only

Supplementary additional file 1: Search strategy

PubMed (2021-05-10)

Concepts	Search strategy keywords	Search	# Results
Community-based programmes	(("community-based" OR "community based" OR community OR complex OR collective) AND (intervention OR program* OR trial OR evaluation OR action OR research)) OR ("Community-Based Participatory Research"[Mesh]) OR "Community Participation"[Mesh]	#1	2,174,786
Social networks	("social network*" OR "social network analysis" OR "social network intervention*" OR "network analysis" OR "community networks" OR "social relationships" OR "social capital" OR "whole networks") OR (("Social Network Analysis"[Mesh] OR "Social Networking"[Mesh]) OR ("Community Networks"[Mesh]))	#2	56,178
Scale-up and sustainability	(("scale-up" OR "scale up" OR sustain* OR implement* OR disseminat*) OR (("Diffusion of Innovation"[Mesh]) OR "Program Evaluation"[Mesh])) OR ("diffusion of innovation" OR "program evaluation")	#3	1,194,227
Non-communicable disease prevention (physical activity and diet)	(Physical activity OR exercise OR diet OR "non communicable" OR chronic OR health promotion OR weight) OR ("Exercise"[Mesh] OR "Diet"[Mesh] OR "Noncommunicable Diseases"[Mesh] OR "Chronic Disease"[Mesh] OR "Health Promotion"[Mesh] OR "Obesity"[Mesh])	#4	4,006,384
Low- and middle-income country setting	((Afghanistan OR Albania OR Algeria OR American Samoa OR Angola OR Armenia OR Azerbaijan OR Bangladesh OR Belarus OR Byelarus OR Belorussia OR Belize OR Benin OR Bhutan OR Bolivia OR Bosnia OR Botswana OR Brazil OR Bulgaria OR Burma OR Burkina Faso OR Burundi OR Cabo Verde OR Cape Verde OR Cambodia OR Cameroon OR Central African Republic OR Chad OR China OR Colombia OR Comoros OR Comores OR Comoro OR Congo OR Costa Rica OR Côte d'Ivoire OR Cuba OR Djibouti OR Dominica OR Dominican Republic OR Ecuador OR Egypt OR El Salvador OR Equatorial Guinea OR Eritrea OR Ethiopia OR Fiji OR Gabon OR Gambia OR Gaza OR Georgia OR Georgia Republic OR Ghana OR Grenada OR Grenadines OR Guatemala OR Guinea OR Guinea-Bissau OR Guyana OR Haiti OR Herzegovina OR Hercegovina OR Honduras OR India OR Indonesia OR Iran OR Iraq OR Ivory Coast OR Jamaica OR Jordan OR Kazakhstan OR Kenya OR Kiribati OR Democratic People's Republic of Korea OR Kosovo OR Kyrgyz OR	#5	6,170,610

For peer review only

	Kirghizia OR Kirghiz OR Kyrgyzstan OR Lao PDR OR Laos OR Lebanon OR Lesotho OR Liberia OR Libya OR Macedonia OR Madagascar OR Malawi OR Malay OR Malaya OR Malaysia OR Maldives OR Mali OR Marshall Islands OR Mauritania OR Mauritius OR Mexico OR Micronesia OR Moldova OR Mongolia OR Montenegro OR Morocco OR Mozambique OR Myanmar OR Namibia OR Nepal OR Nicaragua OR Niger OR Nigeria OR Pakistan OR Palau OR Papua New Guinea OR Paraguay OR Peru OR Philippines OR Principe OR Romania OR Ruanda OR Rwanda OR Samoa OR Sao Tome OR Senegal OR Serbia OR Sierra Leone OR Solomon Islands OR Somalia OR South Africa OR South Sudan OR Sri Lanka OR St Lucia OR St Vincent OR Sudan OR Surinam OR Suriname OR Swaziland OR Syria OR Syrian Arab Republic OR Tajikistan OR Tadzhikistan OR Tajikistan OR Tadzhik OR Tanzania OR Thailand OR Timor OR Togo OR Tonga OR Tunisia OR Turkey OR Turkmen OR Turkmenistan OR Tuvalu OR Uganda OR Ukraine OR Uzbek OR Uzbekistan OR Vanuatu OR Venezuela OR Vietnam OR West Bank OR Yemen OR Zambia OR Zimbabwe) OR (Deprived Countries OR Developing Countries OR Developing Country OR Developing Economies OR Developing Economy OR Developing Nation OR Developing Nations OR Developing World OR LAMI Countries OR LAMI Country OR Less Developed Countries OR Less Developed Country OR Less Developed Economies OR Less Developed Nation OR Less Developed Nations OR Less Developed World OR Lesser Developed Countries OR Lesser Developed Nations OR LMIC OR LMICS OR Low GDP OR Low GNP OR Low Gross Domestic OR Low Gross National OR Low Income OR Lower GDP OR lower gross domestic OR Lower Income OR Middle Income OR Poor Countries OR Poor Country OR Poor Economies OR Poor Economy OR Poor Nation OR Poor Nations OR Poor Population OR Poor Populations OR poor world OR Poorer Countries OR Poorer Economies OR Poorer Economy OR Poorer Nations OR Poorer Population OR Poorer Populations OR Third World OR Transitional Countries OR Transitional Country OR Transitional Economies OR Transitional Economy OR Under Developed Countries OR Under Developed Country OR under developed nations OR Under Developed World OR Under Served Population OR Under Served Populations OR Underdeveloped Countries OR Underdeveloped Country OR underdeveloped economies OR underdeveloped nations OR underdeveloped population OR Underdeveloped World OR Underserved Countries OR Underserved Nations OR Underserved Population OR Underserved Populations)) OR ("Developing Countries"[Mesh]) OR Global South OR Africa OR Latin America OR South America OR Sub-Saharan Africa OR Asia		

For peer review only

Search string	#1 AND #2 AND #3 AND #4 AND #5	#6	584
Limits	Limit to English language; Limit to since year 2000		
Total Result	#1 AND #2 AND #3 AND #4 AND #5 AND limits	#7	556

Scopus (2021-05-10)

Concepts	Search strategy keywords	Search	# Results
Community-based programmes	ALL (("community-based" OR "community based" OR community OR complex OR collective) AND (intervention OR program* OR trial OR evaluation OR action OR research))	#1	12,372,281
Social networks	ALL ("social network*" OR "social network analysis" OR "social network intervention*" OR "network analysis" OR "community networks" OR “social relationships” OR “social capital” OR “whole networks”)	#2	979,911
Scale-up and sustainability	ALL ("scale-up" OR "scale up" OR sustain* OR implement* OR disseminat* OR "diffusion of innovation" OR "program evaluation")	#3	9,528,545
Non-communicable disease prevention (physical activity and diet)	ALL (Physical activity OR exercise OR diet OR "non communicable" OR chronic OR health promotion OR weight)	#4	849,134
Low- and middle-income country setting	ALL ((Afghanistan OR Albania OR Algeria OR “American Samoa” OR Angola OR Armenia OR Azerbaijan OR Bangladesh OR Belarus OR Byelarus OR Belorussia OR Belize OR Benin OR Bhutan OR Bolivia OR Bosnia OR Botswana OR Brazil OR Bulgaria OR Burma OR “Burkina Faso” OR Burundi OR “Cabo Verde” OR “Cape Verde” OR Cambodia OR Cameroon OR “Central African Republic” OR Chad OR China OR Colombia OR Comoros OR Comores OR Comoro OR Congo OR “Costa Rica” OR “Côte d'Ivoire” OR Cuba OR Djibouti OR Dominica OR “Dominican Republic” OR Ecuador OR Egypt OR “El Salvador” OR “Equatorial Guinea” OR Eritrea OR Ethiopia OR Fiji OR Gabon OR Gambia OR Gaza OR Georgia OR “Georgia Republic” OR Ghana OR Grenada OR Grenadines OR Guatemala OR Guinea OR Guinea- Bissau OR Guyana OR Haiti OR Herzegovina OR Hercegovina OR Honduras OR India OR Indonesia OR Iran OR Iraq OR “Ivory Coast” OR Jamaica OR Jordan OR Kazakhstan OR Kenya OR Kiribati OR “Democratic People’s Republic of Korea” OR Kosovo OR Kyrgyz OR Kirghizia OR Kirghiz OR Kyrgyzstan OR Lao PDR OR Laos OR Lebanon OR Lesotho OR Liberia OR Libya OR Macedonia OR Madagascar OR Malawi OR	#5	5,148,871

For peer review only

	Malay OR Malaya OR Malaysia OR Maldives OR Mali OR “Marshall Islands” OR Mauritania OR Mauritius OR Mexico OR Micronesia OR Moldova OR Mongolia OR Montenegro OR Morocco OR Mozambique OR Myanmar OR Namibia OR Nepal OR Nicaragua OR Niger OR Nigeria OR Pakistan OR Palau OR “Papua New Guinea” OR Paraguay OR Peru OR Philippines OR Principe OR Romania OR Ruanda OR Rwanda OR Samoa OR “Sao Tome” OR Senegal OR Serbia OR “Sierra Leone” OR “Solomon Islands” OR Somalia OR “South Africa” OR “South Sudan” OR “Sri Lanka” OR “St Lucia” OR “St Vincent” OR Sudan OR Surinam OR Suriname OR Swaziland OR Syria OR “Syrian Arab Republic” OR Tajikistan OR Tadzhikistan OR Tajikistan OR Tadzhik OR Tanzania OR Thailand OR Timor OR Togo OR Tonga OR Tunisia OR Turkey OR Turkmen OR Turkmenistan OR Tuvalu OR Uganda OR Ukraine OR Uzbek OR Uzbekistan OR Vanuatu OR Venezuela OR Vietnam OR “West Bank” OR Yemen OR Zambia OR Zimbabwe) OR (“Deprived Countries” OR “Developing Countries” OR “Developing Country” OR “Developing Economies” OR “Developing Economy” OR “Developing Nation” OR “Developing Nations” OR “Developing World” OR “LAMI Countries” OR “LAMI Country” OR “Less Developed Countries” OR “Less Developed Country” OR “Less Developed Economies” OR “Less Developed Nation” OR “Less Developed Nations” OR “Less Developed World” OR “Lesser Developed Countries” OR “Lesser Developed Nations” OR LMIC OR LMICS OR “Low GDP” OR “Low GNP” OR “Low Gross Domestic” OR “Low Gross National” OR “Low Income” OR “Lower GDP” OR “lower gross domestic” OR “Lower Income” OR “Middle Income” OR “Poor Countries” OR “Poor Country” OR “Poor Economies” OR “Poor Economy” OR “Poor Nation” OR “Poor Nations” OR “Poor Population” OR “Poor Populations” OR “poor world” OR “Poorer Countries” OR “Poorer Economies” OR “Poorer Economy” OR “Poorer Nations” OR “Poorer Population” OR “Poorer Populations” OR “Third World” OR “Transitional Countries” OR “Transitional Country” OR “Transitional Economies” OR “Transitional Economy” OR “Under Developed Countries” OR “Under Developed Country” OR “under developed nations” OR “Under Developed World” OR “Under Served Population” OR “Under Served Populations” OR “Underdeveloped Countries” OR “Underdeveloped Country” OR “underdeveloped economies” OR “underdeveloped nations” OR “underdeveloped population” OR “Underdeveloped World” OR “Underserved Countries” OR “Underserved Nations” OR “Underserved Population” OR “Underserved Populations”		

For peer review only

	OR "Global South" OR Africa OR "Latin America" OR "South America" OR "Sub-Saharan Africa" OR Asia))		
Search string	#1 AND #2 AND #3 AND #4 AND #5	#6	6,080
Limits	Limit to English language; Limit to since year 2000; Limit document type (Article, Editorial)		
Total Result	#1 AND #2 AND #3 AND #4 AND #5 AND limits	#7	3,211

Web of Science (2021-05-10)

Concepts	Search strategy keywords	Search	# Results
Community-based programmes	ALL=(("community-based" OR "community based" OR community OR complex OR collective) AND (intervention OR program* OR trial OR evaluation OR action OR research))	#1	2,776,017
Social networks	ALL=("social network*" OR "social network analysis" OR "social network intervention*" OR "network analysis" OR "community networks" OR "social relationships" OR "social capital" OR "whole networks")	#2	134,632
Scale-up and sustainability	ALL=("scale-up" OR "scale up" OR sustain* OR implement* OR disseminat* OR "diffusion of innovation" OR "program evaluation")	#3	2,522,901
Non-communicable disease prevention (physical activity and diet)	ALL=(Physical activity OR exercise OR diet OR "non communicable" OR chronic OR health promotion OR weight)	#4	4,314,124
Low- and middle-income country setting	AB=((Afghanistan OR Albania OR Algeria OR "American Samoa" OR Angola OR Armenia OR Azerbaijan OR Bangladesh OR Belarus OR Byelarus OR Belorussia OR Belize OR Benin OR Bhutan OR Bolivia OR Bosnia OR Botswana OR Brazil OR Bulgaria OR Burma OR "Burkina Faso" OR Burundi OR "Cabo Verde" OR "Cape Verde" OR Cambodia OR Cameroon OR "Central African Republic" OR Chad OR China OR Colombia OR Comoros OR Comores OR Comoro OR Congo OR "Costa Rica" OR "Côte d'Ivoire" OR Cuba OR Djibouti OR Dominica OR "Dominican Republic" OR Ecuador OR Egypt OR "El Salvador" OR "Equatorial Guinea" OR Eritrea OR Ethiopia OR Fiji OR Gabon OR Gambia OR Gaza OR Georgia OR "Georgia Republic" OR Ghana OR Grenada OR Grenadines OR Guatemala OR Guinea OR Guinea- Bissau OR Guyana OR Haiti OR Herzegovina OR Hercegovina OR Honduras OR India OR Indonesia OR Iran OR Iraq OR "Ivory Coast" OR Jamaica OR	#5	2,096,577

For peer review only

	Jordan OR Kazakhstan OR Kenya OR Kiribati OR “Democratic People’s Republic of Korea” OR Kosovo OR Kyrgyz OR Kirghizia OR Kirghiz OR Kyrgyzstan OR Lao PDR OR Laos OR Lebanon OR Lesotho OR Liberia OR Libya OR Macedonia OR Madagascar OR Malawi OR Malay OR Malaya OR Malaysia OR Maldives OR Mali OR “Marshall Islands” OR Mauritania OR Mauritius OR Mexico OR Micronesia OR Moldova OR Mongolia OR Montenegro OR Morocco OR Mozambique OR Myanmar OR Namibia OR Nepal OR Nicaragua OR Niger OR Nigeria OR Pakistan OR Palau OR “Papua New Guinea” OR Paraguay OR Peru OR Philippines OR Principe OR Romania OR Ruanda OR Rwanda OR Samoa OR “Sao Tome” OR Senegal OR Serbia OR “Sierra Leone” OR “Solomon Islands” OR Somalia OR “South Africa” OR “South Sudan” OR “Sri Lanka” OR “St Lucia” OR “St Vincent” OR Sudan OR Surinam OR Suriname OR Swaziland OR Syria OR “Syrian Arab Republic” OR Tajikistan OR Tadzhikistan OR Tajikistan OR Tadzhik OR Tanzania OR Thailand OR Timor OR Togo OR Tonga OR Tunisia OR Turkey OR Turkmen OR Turkmenistan OR Tuvalu OR Uganda OR Ukraine OR Uzbek OR Uzbekistan OR Vanuatu OR Venezuela OR Vietnam OR “West Bank” OR Yemen OR Zambia OR Zimbabwe) OR (“Deprived Countries” OR “Developing Countries” OR “Developing Country” OR “Developing Economies” OR “Developing Economy” OR “Developing Nation” OR “Developing Nations” OR “Developing World” OR “LAMI Countries” OR “LAMI Country” OR “Less Developed Countries” OR “Less Developed Country” OR “Less Developed Economies” OR “Less Developed Nation” OR “Less Developed Nations” OR “Less Developed World” OR “Lesser Developed Countries” OR “Lesser Developed Nations” OR LMIC OR LMICS OR “Low GDP” OR “Low GNP” OR “Low Gross Domestic” OR “Low Gross National” OR “Low Income” OR “Lower GDP” OR “lower gross domestic” OR “Lower Income” OR “Middle Income” OR “Poor Countries” OR “Poor Country” OR “Poor Economies” OR “Poor Economy” OR “Poor Nation” OR “Poor Nations” OR “Poor Population” OR “Poor Populations” OR “poor world” OR “Poorer Countries” OR “Poorer Economies” OR “Poorer Economy” OR “Poorer Nations” OR “Poorer Population” OR “Poorer Populations” OR “Third World” OR “Transitional Countries” OR “Transitional Country” OR “Transitional Economies” OR “Transitional Economy” OR “Under Developed Countries” OR “Under Developed Country” OR “under developed nations” OR “Under Developed World” OR “Under Served Population” OR “Under Served Populations” OR “Underdeveloped Countries” OR “Underdeveloped Country” OR “underdeveloped economies” OR “underdeveloped nations” OR “underdeveloped		

For peer review only - http://bmjopen.bmj.com/site/about/guidelines.xhtml

	population” OR “Underdeveloped World” OR “Underserved Countries” OR “Underserved Nations” OR “Underserved Population” OR “Underserved Populations” OR “Global South” OR Africa OR “Latin America” OR “South America” OR “Sub-Saharan Africa” OR Asia))		
Search string	#1 AND #2 AND #3 AND #4 AND #5	#6	172
Limits	Limit to English language; Limit to since year 2000		
Total Result	#1 AND #2 AND #3 AND #4 AND #5 AND limits	#7	162

CINAHL-EBSCO (2021-05-10)

**Concepts**	**Search strategy keywords**	**Search**	**# Results**
Community-based programmes	TX ("community-based" OR "community based" OR community OR complex OR collective) AND (intervention OR program* OR trial OR evaluation OR action OR research)	#1	480,307
Social networks	TX ("social network*" OR "social network analysis" OR "social network intervention*" OR "network analysis" OR "community networks" OR “social relationships” OR “social capital” OR “whole networks”)	#2	37,252
Scale-up and sustainability	TX ("scale-up" OR "scale up" OR sustain* OR implement* OR disseminat* OR "diffusion of innovation" OR "program evaluation")	#3	388,571
Non-communicable disease prevention (physical activity and diet)	TX (Physical activity OR exercise OR diet OR "non communicable" OR chronic OR health promotion OR weight)	#4	1,025,075
Low- and middle-income country setting	TX ((Afghanistan OR Albania OR Algeria OR “American Samoa” OR Angola OR Armenia OR Azerbaijan OR Bangladesh OR Belarus OR Byelarus OR Belorussia OR Belize OR Benin OR Bhutan OR Bolivia OR Bosnia OR Botswana OR Brazil OR Bulgaria OR Burma OR “Burkina Faso” OR Burundi OR “Cabo Verde” OR “Cape Verde” OR Cambodia OR Cameroon OR “Central African Republic” OR Chad OR China OR Colombia OR Comoros OR Comores OR Comoro OR Congo OR “Costa Rica” OR “Côte d'Ivoire” OR Cuba OR Djibouti OR Dominica OR “Dominican Republic” OR Ecuador OR Egypt OR “El Salvador” OR “Equatorial Guinea” OR Eritrea OR Ethiopia OR Fiji OR Gabon OR Gambia OR Gaza OR Georgia OR “Georgia Republic” OR Ghana OR Grenada OR Grenadines OR Guatemala OR Guinea OR Guinea- Bissau OR Guyana OR Haiti OR Herzegovina OR Hercegovina OR	#5	1,199,237

For peer review only

	Honduras OR India OR Indonesia OR Iran OR Iraq OR "Ivory Coast" OR Jamaica OR Jordan OR Kazakhstan OR Kenya OR Kiribati OR "Democratic People's Republic of Korea" OR Kosovo OR Kyrgyz OR Kirghizia OR Kirghiz OR Kyrgyzstan OR Lao PDR OR Laos OR Lebanon OR Lesotho OR Liberia OR Libya OR Macedonia OR Madagascar OR Malawi OR Malay OR Malaya OR Malaysia OR Maldives OR Mali OR "Marshall Islands" OR Mauritania OR Mauritius OR Mexico OR Micronesia OR Moldova OR Mongolia OR Montenegro OR Morocco OR Mozambique OR Myanmar OR Namibia OR Nepal OR Nicaragua OR Niger OR Nigeria OR Pakistan OR Palau OR "Papua New Guinea" OR Paraguay OR Peru OR Philippines OR Principe OR Romania OR Ruanda OR Rwanda OR Samoa OR "Sao Tome" OR Senegal OR Serbia OR "Sierra Leone" OR "Solomon Islands" OR Somalia OR "South Africa" OR "South Sudan" OR "Sri Lanka" OR "St Lucia" OR "St Vincent" OR Sudan OR Surinam OR Suriname OR Swaziland OR Syria OR "Syrian Arab Republic" OR Tajikistan OR Tadzhikistan OR Tajikistan OR Tadzhik OR Tanzania OR Thailand OR Timor OR Togo OR Tonga OR Tunisia OR Turkey OR Turkmen OR Turkmenistan OR Tuvalu OR Uganda OR Ukraine OR Uzbek OR Uzbekistan OR Vanuatu OR Venezuela OR Vietnam OR "West Bank" OR Yemen OR Zambia OR Zimbabwe) OR ("Deprived Countries" OR "Developing Countries" OR "Developing Country" OR "Developing Economies" OR "Developing Economy" OR "Developing Nation" OR "Developing Nations" OR "Developing World" OR "LAMI Countries" OR "LAMI Country" OR "Less Developed Countries" OR "Less Developed Country" OR "Less Developed Economies" OR "Less Developed Nation" OR "Less Developed Nations" OR "Less Developed World" OR "Lesser Developed Countries" OR "Lesser Developed Nations" OR LMIC OR LMICS OR "Low GDP" OR "Low GNP" OR "Low Gross Domestic" OR "Low Gross National" OR "Low Income" OR "Lower GDP" OR "lower gross domestic" OR "Lower Income" OR "Middle Income" OR "Poor Countries" OR "Poor Country" OR "Poor Economies" OR "Poor Economy" OR "Poor Nation" OR "Poor Nations" OR "Poor Population" OR "Poor Populations" OR "poor world" OR "Poorer Countries" OR "Poorer Economies" OR "Poorer Economy" OR "Poorer Nations" OR "Poorer Population" OR "Poorer Populations" OR "Third World" OR "Transitional Countries" OR "Transitional Country" OR "Transitional Economies" OR "Transitional Economy" OR "Under Developed Countries" OR "Under Developed Country" OR "under developed nations" OR "Under Developed World" OR "Under Served Population" OR "Under Served Populations" OR "Underdeveloped Countries" OR "Underdeveloped Country" OR		

For peer review only

	“underdeveloped economies” OR “underdeveloped nations” OR “underdeveloped population” OR “Underdeveloped World” OR “Underserved Countries” OR “Underserved Nations” OR “Underserved Population” OR “Underserved Populations” OR “Global South” OR Africa OR “Latin America” OR “South America” OR “Sub-Saharan Africa” OR Asia))		
Search string	#1 AND #2 AND #3 AND #4 AND #5	#6	274
Limits	Limit to English language; Limit to since year 2000		
Total Result	#1 AND #2 AND #3 AND #4 AND #5 AND limits	#7	258

For peer review only

SocINDEX (2021-05-10)

Concepts	Search strategy keywords	Search	# Results
Community-based programmes	("community-based" OR "community based" OR community OR complex OR collective) AND (intervention OR program* OR trial OR evaluation OR action OR research)	#1	240,457
Social networks	("social network*" OR "social network analysis" OR "social network intervention*" OR "network analysis" OR "community networks" OR “social relationships” OR “social capital” OR “whole networks”)	#2	80,615
Scale-up and sustainability	("scale-up" OR "scale up" OR sustain* OR implement* OR disseminat* OR "diffusion of innovation" OR "program evaluation")	#3	132,008
Non-communicable disease prevention (physical activity and diet)	(Physical activity OR exercise OR diet OR "non communicable" OR chronic OR health promotion OR weight)	#4	76,756
Low- and middle-income country setting	((Afghanistan OR Albania OR Algeria OR “American Samoa” OR Angola OR Armenia OR Azerbaijan OR Bangladesh OR Belarus OR Byelarus OR Belorussia OR Belize OR Benin OR Bhutan OR Bolivia OR Bosnia OR Botswana OR Brazil OR Bulgaria OR Burma OR “Burkina Faso” OR Burundi OR “Cabo Verde” OR “Cape Verde” OR Cambodia OR Cameroon OR “Central African Republic” OR Chad OR China OR Colombia OR Comoros OR Comores OR Comoro OR Congo OR “Costa Rica” OR “Côte d'Ivoire” OR Cuba OR Djibouti OR Dominica OR “Dominican Republic” OR Ecuador OR Egypt OR “El Salvador” OR “Equatorial Guinea” OR Eritrea OR Ethiopia OR Fiji OR Gabon OR Gambia OR Gaza OR Georgia OR “Georgia Republic” OR Ghana OR Grenada OR Grenadines OR Guatemala OR Guinea OR Guinea-	#5	428,329

	Bissau OR Guyana OR Haiti OR Herzegovina OR Hercegovina OR Honduras OR India OR Indonesia OR Iran OR Iraq OR "Ivory Coast" OR Jamaica OR Jordan OR Kazakhstan OR Kenya OR Kiribati OR "Democratic People's Republic of Korea" OR Kosovo OR Kyrgyz OR Kirghizia OR Kirghiz OR Kyrgyzstan OR Lao PDR OR Laos OR Lebanon OR Lesotho OR Liberia OR Libya OR Macedonia OR Madagascar OR Malawi OR Malay OR Malaya OR Malaysia OR Maldives OR Mali OR "Marshall Islands" OR Mauritania OR Mauritius OR Mexico OR Micronesia OR Moldova OR Mongolia OR Montenegro OR Morocco OR Mozambique OR Myanmar OR Namibia OR Nepal OR Nicaragua OR Niger OR Nigeria OR Pakistan OR Palau OR "Papua New Guinea" OR Paraguay OR Peru OR Philippines OR Principe OR Romania OR Ruanda OR Rwanda OR Samoa OR "Sao Tome" OR Senegal OR Serbia OR "Sierra Leone" OR "Solomon Islands" OR Somalia OR "South Africa" OR "South Sudan" OR "Sri Lanka" OR "St Lucia" OR "St Vincent" OR Sudan OR Surinam OR Suriname OR Swaziland OR Syria OR "Syrian Arab Republic" OR Tajikistan OR Tadzhikistan OR Tajikistan OR Tadzhik OR Tanzania OR Thailand OR Timor OR Togo OR Tonga OR Tunisia OR Turkey OR Turkmen OR Turkmenistan OR Tuvalu OR Uganda OR Ukraine OR Uzbek OR Uzbekistan OR Vanuatu OR Venezuela OR Vietnam OR "West Bank" OR Yemen OR Zambia OR Zimbabwe) OR ("Deprived Countries" OR "Developing Countries" OR "Developing Country" OR "Developing Economies" OR "Developing Economy" OR "Developing Nation" OR "Developing Nations" OR "Developing World" OR "LAMI Countries" OR "LAMI Country" OR "Less Developed Countries" OR "Less Developed Country" OR "Less Developed Economies" OR "Less Developed Nation" OR "Less Developed Nations" OR "Less Developed World" OR "Lesser Developed Countries" OR "Lesser Developed Nations" OR LMIC OR LMICS OR "Low GDP" OR "Low GNP" OR "Low Gross Domestic" OR "Low Gross National" OR "Low Income" OR "Lower GDP" OR "lower gross domestic" OR "Lower Income" OR "Middle Income" OR "Poor Countries" OR "Poor Country" OR "Poor Economies" OR "Poor Economy" OR "Poor Nation" OR "Poor Nations" OR "Poor Population" OR "Poor Populations" OR "poor world" OR "Poorer Countries" OR "Poorer Economies" OR "Poorer Economy" OR "Poorer Nations" OR "Poorer Population" OR "Poorer Populations" OR "Third World" OR "Transitional Countries" OR "Transitional Country" OR "Transitional Economies" OR "Transitional Economy" OR "Under Developed Countries" OR "Under Developed Country" OR "under developed nations" OR "Under Developed World" OR "Under Served Population" OR "Under Served Populations" OR "Underdeveloped Countries" OR "Underdeveloped		

For peer review only

	Country" OR "underdeveloped economies" OR "underdeveloped nations" OR "underdeveloped population" OR "Underdeveloped World" OR "Underserved Countries" OR "Underserved Nations" OR "Underserved Population" OR "Underserved Populations" OR "Global South" OR Africa OR "Latin America" OR "South America" OR "Sub-Saharan Africa" OR Asia))		
Search string	#1 AND #2 AND #3 AND #4 AND #5	#6	32
Limits	Limit to English language; Limit to since year 2000		
Total Result	#1 AND #2 AND #3 AND #4 AND #5 AND limits	#7	29

Cochrane (2021-05-10)

**Concepts**	**Search strategy keywords**	**Search**	**# Results**
Community-based programmes	("community-based" OR "community based" OR community OR complex OR collective) AND (intervention OR program* OR trial OR evaluation OR action OR research)<br>AND MeSH descriptor: [Community-Based Participatory Research] explode all trees	#1	86,794
Social networks	("social network*" OR "social network analysis" OR "social network intervention*" OR "network analysis" OR "community networks" OR "social relationships" OR "social capital" OR "whole networks")<br>AND MeSH descriptor: [Social Network Analysis] explode all trees	#2	1,514
Scale-up and sustainability	("scale-up" OR "scale up" OR sustain* OR implement* OR disseminat* OR "diffusion of innovation" OR "program evaluation")<br>AND MeSH descriptor: [Program Evaluation] explode all trees	#3	105,571
Non-communicable disease prevention (physical activity and diet)	(Physical activity OR exercise OR diet OR "non communicable" OR chronic OR health promotion OR weight)<br>AND MeSH descriptor: [Noncommunicable Diseases] explode all trees	#4	326,399
Low- and middle-income country setting	Not able to complete on this database		–
Search string	#1 AND #2 AND #3 AND #4	#5	304
Limits	Limit to exclude protocols; Limit to since year 2000		
Total Result	#1 AND #2 AND #3 AND #4 AND limits	#6	268

For peer review only

International Bibliography of the Social Sciences (2021-05-10)

Concepts	Search strategy keywords	Search	# Results
Community-based programmes	Ab(("community-based" OR "community based" OR community OR complex OR collective) AND (intervention OR program* OR trial OR evaluation OR action OR research))	#1	108,621
Social networks	Ab("social network*" OR "social network analysis" OR "social network intervention*" OR "network analysis" OR "community networks" OR “social relationships” OR “social capital” OR “whole networks”)	#2	29,987
Scale-up and sustainability	Ab("scale-up" OR "scale up" OR sustain* OR implement* OR disseminat* OR "diffusion of innovation" OR "program evaluation")	#3	186,317
Non-communicable disease prevention (physical activity and diet)	Anywhere (Physical activity OR exercise OR diet OR "non communicable" OR chronic OR health promotion OR weight)	#4	378,734
Low- and middle-income country setting	Anywhere ((Afghanistan OR Albania OR Algeria OR “American Samoa” OR Angola OR Armenia OR Azerbaijan OR Bangladesh OR Belarus OR Byelarus OR Belorussia OR Belize OR Benin OR Bhutan OR Bolivia OR Bosnia OR Botswana OR Brazil OR Bulgaria OR Burma OR “Burkina Faso” OR Burundi OR “Cabo Verde” OR “Cape Verde” OR Cambodia OR Cameroon OR “Central African Republic” OR Chad OR China OR Colombia OR Comoros OR Comores OR Comoro OR Congo OR “Costa Rica” OR “Côte d'Ivoire” OR Cuba OR Djibouti OR Dominica OR “Dominican Republic” OR Ecuador OR Egypt OR “El Salvador” OR “Equatorial Guinea” OR Eritrea OR Ethiopia OR Fiji OR Gabon OR Gambia OR Gaza OR Georgia OR “Georgia Republic” OR Ghana OR Grenada OR Grenadines OR Guatemala OR Guinea OR Guinea- Bissau OR Guyana OR Haiti OR Herzegovina OR Hercegovina OR Honduras OR India OR Indonesia OR Iran OR Iraq OR “Ivory Coast” OR Jamaica OR Jordan OR Kazakhstan OR Kenya OR Kiribati OR “Democratic People’s Republic of Korea” OR Kosovo OR Kyrgyz OR Kirghizia OR Kirghiz OR Kyrgyzstan OR Lao PDR OR Laos OR Lebanon OR Lesotho OR Liberia OR Libya OR Macedonia OR Madagascar OR Malawi OR Malay OR Malaya OR Malaysia OR Maldives OR Mali OR “Marshall Islands” OR Mauritania OR Mauritius OR Mexico OR Micronesia OR Moldova OR Mongolia OR Montenegro OR Morocco OR Mozambique OR Myanmar OR Namibia OR Nepal OR Nicaragua OR Niger OR Nigeria OR Pakistan OR Palau OR “Papua New Guinea” OR	#5	1,602,554

For peer review only

	Paraguay OR Peru OR Philippines OR Principe OR Romania OR Ruanda OR Rwanda OR Samoa OR "Sao Tome" OR Senegal OR Serbia OR "Sierra Leone" OR "Solomon Islands" OR Somalia OR "South Africa" OR "South Sudan" OR "Sri Lanka" OR "St Lucia" OR "St Vincent" OR Sudan OR Surinam OR Suriname OR Swaziland OR Syria OR "Syrian Arab Republic" OR Tajikistan OR Tadzhikistan OR Tajikistan OR Tadzhik OR Tanzania OR Thailand OR Timor OR Togo OR Tonga OR Tunisia OR Turkey OR Turkmen OR Turkmenistan OR Tuvalu OR Uganda OR Ukraine OR Uzbek OR Uzbekistan OR Vanuatu OR Venezuela OR Vietnam OR "West Bank" OR Yemen OR Zambia OR Zimbabwe) OR ("Deprived Countries" OR "Developing Countries" OR "Developing Country" OR "Developing Economies" OR "Developing Economy" OR "Developing Nation" OR "Developing Nations" OR "Developing World" OR "LAMI Countries" OR "LAMI Country" OR "Less Developed Countries" OR "Less Developed Country" OR "Less Developed Economies" OR "Less Developed Nation" OR "Less Developed Nations" OR "Less Developed World" OR "Lesser Developed Countries" OR "Lesser Developed Nations" OR LMIC OR LMICS OR "Low GDP" OR "Low GNP" OR "Low Gross Domestic" OR "Low Gross National" OR "Low Income" OR "Lower GDP" OR "lower gross domestic" OR "Lower Income" OR "Middle Income" OR "Poor Countries" OR "Poor Country" OR "Poor Economies" OR "Poor Economy" OR "Poor Nation" OR "Poor Nations" OR "Poor Population" OR "Poor Populations" OR "poor world" OR "Poorer Countries" OR "Poorer Economies" OR "Poorer Economy" OR "Poorer Nations" OR "Poorer Population" OR "Poorer Populations" OR "Third World" OR "Transitional Countries" OR "Transitional Country" OR "Transitional Economies" OR "Transitional Economy" OR "Under Developed Countries" OR "Under Developed Country" OR "under developed nations" OR "Under Developed World" OR "Under Served Population" OR "Under Served Populations" OR "Underdeveloped Countries" OR "Underdeveloped Country" OR "underdeveloped economies" OR "underdeveloped nations" OR "underdeveloped population" OR "Underdeveloped World" OR "Underserved Countries" OR "Underserved Nations" OR "Underserved Population" OR "Underserved Populations" OR "Global South" OR Africa OR "Latin America" OR "South America" OR "Sub-Saharan Africa" OR Asia))		
Search string	#1 AND #2 AND #3 AND #4 AND #5	#6	2,727
Limits	Limit to English language; Limit to since year 2000; Limit to exclude ("Dissertations & Theses" OR "Conference Papers & Proceedings" OR "Books" OR "Working Papers")		

For peer review only - http://bmjopen.bmj.com/site/about/guidelines.xhtml

Total Result	#1 AND #2 AND #3 AND #4 AND #5 AND limits	#7	1,590

Journal and Google basic search (2021-05-10)

**Concepts**	**Search strategy keywords**	**Search**	**# Results**
Social Networks	("scale-up" OR "sustainability" OR "implementation") AND ("physical activity" OR "diet" OR "chronic" OR "non-communicable" OR health promotion OR weight), since 2000, excl. books	#1	246
Applied Network Science	("scale-up" OR "sustainability" OR "implementation") AND ("physical activity" OR "diet" OR "chronic" OR "non-communicable" OR health promotion OR weight), since 2000, excl. books	#1	31
Journal of Social Structure	("scale-up" OR "sustainability" OR "implementation") AND ("physical activity" OR "diet" OR "chronic" OR "non-communicable" OR health promotion OR weight), since 2000, excl. books	#1	3
Google Scholar (UK based)	("social network" OR "network analysis" OR "social capital" OR "social relationships" AND ("scale up" OR sustain OR implement) AND ("physical activity" OR diet OR chronic OR "non communicable") AND ("low income" OR "middle income" OR LMIC), since 2000	First 3 pages	30
Google (UK based)	("social network" OR "network analysis" OR "social capital" OR "social relationships") AND ("scale up" OR sustain OR implement) AND ("physical activity" OR diet OR chronic OR "non communicable") AND ("low income" OR "middle income" OR LMIC), since 2000	First 3 pages	30

For peer review only
